# Methods guiding stakeholder engagement in planning a pragmatic study on changing stroke systems of care

**DOI:** 10.1017/cts.2016.26

**Published:** 2017-02-27

**Authors:** Sabina B. Gesell, Karen Potvin Klein, Jacqueline Halladay, Janet Prvu Bettger, Janet Freburger, Doyle M. Cummings, Barbara J. Lutz, Sylvia Coleman, Cheryl Bushnell, Wayne Rosamond, Pamela W. Duncan

**Affiliations:** 1 Department of Social Sciences and Health Policy, Wake Forest School of Medicine, Winston-Salem, NC, USA; 2 Clinical and Translational Science Institute, Wake Forest School of Medicine, Winston-Salem, NC, USA; 3 Department of Family Medicine, University of North Carolina School of Medicine at Chapel Hill, Chapel Hill, NC, USA; 4 School of Nursing, Duke University, Durham, NC, USA; 5 Department of Physical Therapy, School of Health and Rehabilitation Sciences, University of Pittsburgh, Pittsburgh, PA, USA; 6 Departments of Family Medicine and Public Health, Brody School of Medicine, East Carolina University, Greenville, NC, USA; 7 School of Nursing, University of North Carolina Wilmington, Wilmington, NC, USA; 8 Department of Neurology, Wake Forest School of Medicine, Winston-Salem, NC, USA; 9 Wake Forest Baptist Stroke Center, Wake Forest Baptist Medical Center, Winston-Salem, NC, USA; 10 Department of Epidemiology, Gillings School of Global Public Health, University of North Carolina at Chapel Hill, Chapel Hill, NC, USA; 11 Department of Neurology, Wake Forest School of Medicine, Winston-Salem, NC, USA

**Keywords:** pragmatic clinical trial, comparative effectiveness research, patient engagement, community-based participatory research, stroke

## Abstract

Background: The Comprehensive Post-Acute Stroke Services (COMPASS) Study is one of the first large pragmatic randomized-controlled clinical trials using comparative effectiveness research methods, funded by the Patient-Centered Outcomes Research Institute. In the COMPASS Study, we compare the effectiveness of a patient-centered, transitional care intervention versus usual care for stroke patients discharged home from acute care. Outcomes include stroke patient post-discharge functional status and caregiver strain 90 days after discharge, and hospital readmissions. A central tenet of Patient-Centered Outcomes Research Institute-funded research is stakeholder engagement throughout the research process. However, evidence on how to successfully implement a pragmatic trial that changes systems of care in combination with robust stakeholder engagement is limited. This combination is not without challenges. Methods: We present our approach for broad-based stakeholder engagement in the context of a pragmatic trial with the participation of patients, caregivers, community stakeholders, including the North Carolina Stroke Care Collaborative hospital network, and policy makers. To maximize stakeholder engagement throughout the COMPASS Study, we employed a conceptual model with the following components: (1) Patient and Other Stakeholder Identification and Selection; (2) Patient and Other Stakeholder Involvement Across the Spectrum of Research Activities; (3) Dedicated Resources for Patient and Other Stakeholder Involvement; (4) Support for Patient and Other Stakeholder Engagement Through Organizational Processes; (5) Communication with Patients and Other Stakeholders; (6) Transparent Involvement Processes; (7) Tracking of Engagement; and (8) Evaluation of Engagement. Conclusion: In this paper, we describe how each component of the model is being implemented and how this approach addresses existing gaps in the literature on strategies for engaging stakeholders in meaningful and useful ways when conducting pragmatic trials.

## Introduction

Engaging patients and other stakeholders in the continuum of biomedical research is a promising approach to creating more effective interventions and accelerating translation of effective interventions into practice [1–6]. Stakeholder engagement is a collaborative approach to research that values the unique perspectives and strengths of nontraditional research partners. It shares the spirit and goals of community-based participatory research (CBPR), but does not require the full model of CBPR as a starting point. Like CBPR, stakeholder-engaged research blends lived experiences of patients, expertise of service providers, power of policy makers, and rigorous science. The focus is on conducting relevant research, informing policy, and promoting successful implementation and long-term sustainability.

In parallel, comparative effectiveness research is the direct comparison of 2 or more interventions to determine which works best for which patients. When designed to evaluate the effectiveness of interventions in real-world practice with heterogeneous patient populations, the study is a pragmatic clinical trial [7].

One of the first 5 large pragmatic clinical trials funded by the Patient-Centered Outcomes Research Institute (PCORI) is the Comprehensive Post-Acute Stroke Services (COMPASS) Study. COMPASS will randomize 41 hospitals (40 randomized units) and enroll a widely diverse group of 6000 patients across North Carolina (NC). Results of the trial will provide evidence to create national quality standards for post-acute stroke care delivery, which currently do not exist.

The purpose of the COMPASS Study is to evaluate the comparative effectiveness of a patient-centered, transitional care model that provides both structure and processes for post-acute care of stroke patients versus usual care. Our working hypothesis is that the patient-centered model will improve functional outcomes and reduce caregiver strain and hospital readmissions.

PCORI has established stakeholder engagement as a central tenet of their funded research portfolio. There is currently little guidance in the biomedical literature on how best to create and foster stakeholder engagement throughout the research process [8–10]. The gaps include a lack of understanding of how to engage a broad range of critical decision makers across the health-care system; which methods and modes of stakeholder engagement are feasible during the earlier and later phases of research; how the views of stakeholders are synthesized and used to shape research design, implementation, and dissemination; and how to partner with stakeholders to increase intervention effects, reduce disparities, and/or sustain tested interventions [10, 11]. Thus, the purpose of this article is to address gaps in the literature on stakeholder engagement in comparative effectiveness research by presenting the model that guides our stakeholder engagement efforts, and sharing the methods we used throughout the pre-award and post-award study design periods to inform best practice methods for engaging stakeholders in large pragmatic trials.

## Methods

### Overview

The COMPASS intervention is built on early supported discharge (ESD) studies in Canada and Europe [12]. ESD is now standard of care in the United Kingdom [13] and Canada [14], but has not been used or evaluated in the US or rural settings. The COMPASS model of care combines ESD with Centers for Medicare and Medicaid Services (CMS) transitional care services provided by advanced practice providers (APP) and ESD services coordinated by the APPs and adapted for the US health-care system.

The COMPASS intervention provides both structure and processes to post-acute care for stroke patients. The innovative intervention combines services from a post-acute care coordinator (PAC) who is an registered nurse, and an APP (physician assistant (PA) or nurse practitioner (NP)) with linkages to community-based services to enhance continuity and coordination of stroke post-acute care. Patients are contacted at discharge, 2 days after discharge by phone, an in-person visit within 7–14 days after discharge, and phone calls at 30 and 60 days after discharge. During the clinic visit within 7–14 days of discharge, the PAC performs standardized assessments to assess the physical, social, and medical determinants of health and specifically screens for common post-stroke problems. The COMPASS intervention is unique in its focus on addressing post-discharge needs of both patients and family caregivers. An assessment of the patient’s functional status and related post-acute care needs, and the caregiver’s capacity for assisting the patient provide the basis for an individualized electronic care plan addressing specific needs; linking patients and caregivers to relevant community-based services (eg, caregiver support, medication management), and follow-up care. The PA or NP reviews the results of each assessment with the PAC and—incorporating input from the patient and caregiver—creates an individualized electronic care plan for each patient. This plan is shared with home health, outpatient therapists, primary care, and community service providers. Therefore, all providers—and the patient and caregiver—understand and have reviewed the plan of care. The effectiveness of the intervention is assessed via a 90-day outcome survey call to the patient, mail survey to the caregiver, and claims data.

Research reported in this manuscript was funded through a PCORI Award. PCORI had no role in the design of the study, data collection or interpretation, or writing or approving the finished manuscript.

### Conceptual Model of Engagement

In designing our stakeholder engagement model and processes, we began with the engagement standards proposed by Curtis *et al*. [15] in their report to PCORI’s Methodology Committee. Given the complexity of a large pragmatic trial, we expanded this rubric to include: (1) Patient and Other Stakeholder Identification and Selection; (2) Patient and Other Stakeholder Involvement Across the Spectrum of Research Activities; (3) Dedicated Resources for Patient and Other Stakeholder Involvement; (4) Support for Patient and Other Stakeholder Engagement Through Organizational Processes; (5) Communication with Patients and Other Stakeholders; (6) Transparent Involvement Processes; (7) Tracking of Engagement; and (8) Evaluation of Engagement. Below we describe each model component and how it was operationalized.

#### Patient and Other Stakeholder Identification and Selection

Our stakeholder partners are listed in Table 1. COMPASS team members have partnered for years with many groups conducting projects focused on hyper-acute and acute stroke care in NC. The NC Stroke Care Collaborative [16, 17], originally funded as a Paul Coverdell National Acute Stroke Registry by the Centers for Disease Control and Prevention, was established in 2004 and provides the COMPASS Study infrastructure. The Collaborative had partnered with the American Heart Association/American Stroke Association’s Get With The Guidelines^®^ Stroke program to improve quality of acute care. The academic centers that came together to create COMPASS had also worked with the NC Department of Health and Human Services and the Justus-Warren Heart Disease and Stroke Prevention Task Force on stroke research and improving systems of care. The current COMPASS project capitalized on extant systems and relationships around stroke systems of care in NC and expanded their focus from the acute care setting to the post-discharge setting.

**Table 1:** Stakeholder groups engaged in the Comprehensive Post-Acute Stroke Services (COMPASS) Study

Stakeholder groups	Rationale for inclusion
Patients	
Diversity in race, sex, SES, age, rural/urban	The most knowledgeable about how usual care is failing them during recovery from stroke. Given the profound inequalities in stroke outcomes, we are intentionally promoting health equity by including the voices of female, African-American, and rural North Carolinians
Family caregivers	
Diversity in race, sex, SES, age, rural/urban	Caregiver support is one of the most important factors in a stroke patient’s recovery. They are the most knowledgeable about how usual care is failing patients and their caregivers
Clinicians	
Primary care (rural/urban)	They are caring for patients after discharge and without them we cannot move population health on stroke forward
Pharmacy (rural/urban)	Patients, neurologists, and primary care physicians all identified medication management as a modifiable risk factor
Home health agencies (rural/urban)	Bayada Home Health, Gentiva, and Kindred are the state’s leading purveyors of skilled nursing services and home-based therapies (eg, physical therapy, occupational therapy, speech therapy) for improving health, regaining independence, and becoming self-sufficient as patients transition back home and into their communities after major illness or injury
Outpatient physical, occupational, and speech therapy	They are essential for carrying out the care plan for recovery for stroke patients. They also have the most face-to-face time with stroke patients and can provide reinforcement of the key messages of COMPASS
Hospital stroke team (rural/urban) Neurologists Stroke nurse practitioners Nurses Stroke care coordinators Therapists (physical, occupational, speech)	The multidisciplinary members of the hospital stroke team are essential for identifying eligible stroke patients and developing the discharge care plans for these patients. Although the majority of the intervention occurs in the post-discharge setting, case ascertainment and the initiation of the intervention begins before discharge. These stakeholders also need to be aware of the information given to patients on behalf of the study
Community-based services	
Piedmont Triad Regional Council Area Agency on Aging (PTRC AAA)	The AAA network manages the allocation of federal and state funding to the community-based services that stroke patients and their caregivers need—but often cannot quickly access—in recovery (eg, transportation services, home modifications, caregiver support). By partnering closely with the PTRC AAA we gained access to all AAAs in NC
CareNet Counseling	Network of community-based spiritually integrated counseling providers across the state. Depression is common after stroke but undertreated, often because mental health services are difficult to access in rural NC. This is an alternative source for behavioral health support services that are critical to chronic care management of stroke
FaithHealthNC	FaithHealthNC is a partnership between congregations and health systems in NC. FaithHealthNC staff train volunteers from congregations and the community to offer health-care ministries to anyone in their community who is in need. They provide support before, during, and after hospitalization (eg, make home visits, provide emotional and spiritual support, and help with meals, transportation, medications, and other needs). This network of congregations can provide some of the services the NCAAA and NCDHHS cannot due to restricted funding
Hospitals and health systems	
Clinical informatics experts	IT stakeholders are needed to be aware of the minimum IT requirements for COMPASS to function properly in the clinic. These stakeholders will be essential for ensuring that wireless technology is available to use the electronic care plan website, both on PC or laptop, and on the iPad. Also, these connections need to be firewall protected
Quality and systems improvement experts	COMPASS is based on the use of quality improvement methods to ensure the workflow and processes are constantly being evaluated and improved upon. The quality departments and leaders are needed to ensure COMPASS is included in the list of quality initiatives for that hospital
NC Stroke Care Collaborative network of hospitals	An existing network of hospitals dedicated to improving acute stroke care; originally funded as a Paul Coverdell National Acute Stroke Registry by the Centers for Disease Control and Prevention in 2004, provided the initial COMPASS Study infrastructure
Regional NC Stroke Networks	Regional stroke networks across the state include hospitals that are receiving stroke education and updates on the latest in stroke care. COMPASS is including these stakeholders because their yearly meetings are a convenient forum for updating these participants on COMPASS
Training institutions	
Northwest Area Health Education Center (NW AHEC), NC	Has existing infrastructure to train health-care providers across the state. By partnering closely with the Northwest AHEC, NC we gained access to all AHECs in NC
East Carolina University Center for Health Disparities	Expertise in tailoring health messages and materials for special populations
Payer	
NC chapter of a national health insurance company	Expressed interest in using study results for benefit design which has potential to support sustainability of the intervention
Policy maker	
American Heart Association/American Stroke Association	It has the largest national and international footprint to disseminate study results to both the public and the medical community (if warranted)
Justus-Warren Heart Disease and Stroke Prevention Task Force	It includes legislators and other stroke champions and key stakeholders that can inform, recommend, and support critically important policy changes at the state level that facilitate improved stroke care, including assuring funding to hospitals
NC Stroke Advisory Council	This body informs the legislative Justus-Warren Heart Disease and Stroke Prevention Task Force including providing findings and recommendations for development and maintenance of NC’s Stroke System of Care
NC Department of Health and Human Services	The Community and Clinical Connections for Prevention and Health Branch supports communities in implementing evidence-based programs relevant to stroke recovery (eg, Check, Change, Control program for hypertension management, Diabetes Prevention Program, and Diabetes Education Recognition Program for diabetes self-management education and support), and acts as a key resource for patient-facing and caregiver-facing materials (eg, disseminating user-friendly video on how to accurately check blood pressure at home, and various medication assistance programs) that are critical to recovery after stroke
NC Stroke Association (NCSA)	The NCSA is committed to addressing the fundamental barrier to timely stroke treatment in rural and underserved NC counties by funding hospitals to become Acute Stroke Ready. It provides programs to hospitals across the state for improving stroke care, especially with respect to stroke risk factor screenings, and post-discharge follow-up. These programs will continue in parallel with COMPASS. In addition, the NCSA can be a resource for dissemination of COMPASS following the trial

SES, socio-economic status; NC, North Carolina; NCDHHS, NC Department of Health and Human Services; IT, information technology; eCare Plan, electronic care plan; PC, personal computer; NCSA, NC Stroke Association.

While preparing the PCORI proposal, we identified stakeholder partners from among those listed above, who actively participated in the study planning and grant writing. Still more stakeholders were added after funding was awarded, primarily from the study team’s professional networks. These key stakeholders have established working relationships with the study team. Importantly, they were enthusiastic about joining the project and did not require a lengthy pre-engagement period to agree to participate. Prompt participation of stakeholders was essential to meet the rapid turnaround time between PCORI’s acceptance of the COMPASS letter of intent and the deadline for the full proposal. We later expanded the number of partners during study design phase, seeking to include all stakeholder groups who would be affected by the study and could influence its success. COMPASS now includes all project-relevant stakeholder groups whose input has shaped intervention design and implementation plans, for the purpose of maximizing effectiveness and uptake.

The academic members of the COMPASS team represent 5 major universities in NC with expertise in neurology, nursing, rehabilitation, pharmacy, primary care, public health, health services research, quality improvement, epidemiology, and biostatistics. Importantly, they also have decades of experience engaging stakeholders in health services research, health system change, and policy change.

We included thought leaders in each stakeholder group, including state policy makers, to support dissemination and long-term sustainability. A previous systematic review of stakeholder engagement in research indicated that engagement with patients was frequent, whereas engagement with clinicians and other stakeholders like payers, product makers, and policy makers was not [10]. In COMPASS, we have included a broad range of critical decision makers across the health-care system and community to maximally support patients’ recovery after stroke (see Table 1).

Importantly, we sought the participation of a racially diverse group of patient partners who share lived, but varied, experiences of stroke recovery. We also wanted to be guided by people with experiences in advocacy and hospital politics who understood the communities in rural eastern NC, where the risk for stroke is the highest in the nation. The lead patient partners for COMPASS are a female African-American stroke survivor known in NC as an effective advocate and patient leader in initiatives for stroke and heart disease prevention and a male Caucasian stroke survivor who, after his recovery, became involved in his local hospital and is now vice chair of a hospital board in eastern NC. These choices are important because African-Americans, women, and residents of the eastern part of the state are at greater risk of poor outcomes after a stroke and their voices needed to be incorporated into the COMPASS Study.

#### Patient and Other Stakeholder Involvement Across the Spectrum of Research Activities

We embedded patients and other stakeholders in the COMPASS Steering Committee and all study subgroups according to their self-reported interests and skills, so they have input and support decision making across the research process. Stakeholders experience the iterative nature of research and their input is incorporated into research decisions. Examples include educating data collectors to slowly read survey questions so stroke survivors can understand them, selecting outcome measures, and making the consent process understandable. This diffusion of stakeholder influence rests on shared leadership and facilitates management of stakeholder engagement for a trial of this size and complexity.

A challenge of engaging a large and diverse number of stakeholders across sectors and across a wide geographic area is that not all stakeholders interact with all investigators. However, by embedding stakeholder partners in all subgroups, researchers and stakeholders can maintain close working relationships around the topics of their stated preferences. Stakeholders’ self-reported interests, expertise, time, and communication requirements informed who would work on which tasks. We consciously allow the level and type of engagement to vary by stakeholder according to interest, skill, time, health status, project needs, and budget constraints. We anticipate that these strategies will leverage stakeholders’ strengths and interests and foster more meaningful and productive participation.

**Fig. 1:**
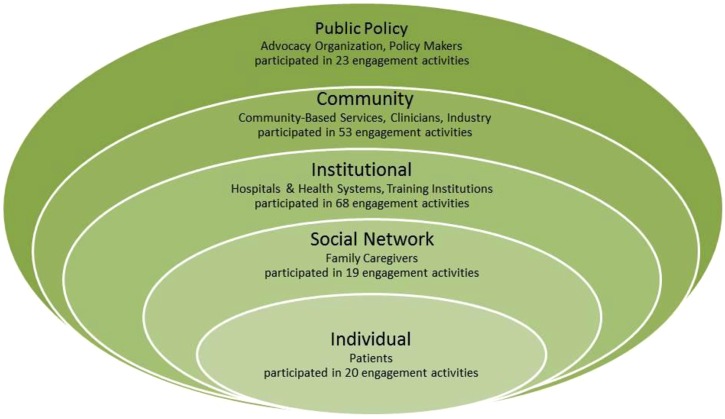
Stakeholder groups that participated in the planning of the Comprehensive Post-Acute Stroke Services (COMPASS) study.

During the study planning/design phase we included stakeholders in 136 engagement activities, 18 of which occurred during proposal writing or before PCORI funding was awarded, 118 within the first 10 months of the grant. Using PCORI’s definition of engagement levels, of these 136 engagement activities, 66 (48%) were information sharing, 40 (30%) were consultation, 17 (12%) were collaboration, and 13 (10%) were stakeholder directed. Engagement also cut across levels of influence (Fig. 1).

To illustrate, multiple stakeholders from multiple groups defined intervention goals, objectives, and key messages; and developed intervention materials and activities based on the existing scientific evidence [12, 18, 19], payment structures, clinical workflow, and stroke patients’, caregivers’, and clinicians’ lived experiences. Multiple stakeholder groups also determined intervention-specific roles, and the most effective training methods for these various roles given existing time constraints. These groups also developed intervention training modules and materials and will train hospital-based and community-based providers. Patient and caregiver feedback was used to determine our selection of outcomes and data collection procedures.

Patients and caregivers also shaped our consent process for data collection. For example, our partners reviewed several iterations of the consent form to retain Institutional Review Board (IRB)-required content that will (1) foster comprehension among potential participants with a range of health literacy levels, and (2) not raise concerns among potential control group participants (receiving usual care) because they will not receive the intervention. The result is a consent protocol for pragmatic trials that meets IRB requirements, but will be easier to understand and be received favorably by potential participants.

Community-based service providers assisted with hospital and patient recruitment by leveraging their professional and personal connections to key decision makers within hospitals who made the decisions about enrolling their hospitals or hospital systems in the trial.

Hospital-based stakeholders included stroke program coordinators, stroke neurologists, physician emergency department directors, quality/performance improvement coordinators, nurse managers, patient education directors, and senior hospital administrators. These stakeholders made a key contribution to the study design by advocating for a delayed start design [20], meaning that, at the end of the trial, all hospitals enrolled in the study eventually will receive the intervention. At baseline, hospitals are randomized to either administer the COMPASS intervention or to provide their usual standard of care (control). In phase 1, patients in these hospitals are followed for 90 days so that the effects of the intervention on outcomes can be observed. In phase 2, all hospitals implement the COMPASS intervention, and the data obtained during this phase will be used to evaluate how well the intervention hospitals sustained the intervention without grant support.

Our statistical collaborators agreed that this design provides the best statistical control to measure the intervention’s effects, and our hospital stakeholders agreed that this design would help ensure that all communities benefit from study participation, which was essential to generate buy-in from individual hospitals and larger health systems.

Although some stakeholder partners contribute to the COMPASS Study with limited financial compensation (patients, caregivers, Piedmont Triad Regional Council Area Agency on Aging), most do not. Several patients and caregivers decline payment because they value “giving back” to their communities and being involved in a state-wide study aimed at improving the way we deliver care to stroke patients. Other stakeholders recognize value in their participation in other ways. Examples include study participation facilitating entry into hospital systems to build previously inaccessible partnerships and access to study team members, who can advise stakeholder organizations on survey design or program design unrelated to stroke care. Building such relationships is time consuming, but making them a priority holds mutual benefits and sows the seeds of long-lasting partnerships.

#### Dedicated Resources for Patient and Other Stakeholder Involvement

The more stakeholders are engaged, the more feedback is received. This can lead to still more relationships, more feedback, and more management. In the COMPASS Study, a faculty “engagement officer” (25% effort) is charged with developing and executing a strategy to weave engagement through the trial and holding the study team accountable. A research coordinator (100% effort) is dedicated to engagement efforts. The oversight of engagement activities is centralized, but all faculty and staff team members (except biostatisticians) spend time leading engagement activities in study subgroups (eg, intervention, outcomes measurement). COMPASS project managers can also direct additional administrative staff to support engagement efforts when needed. The engagement and administrative teams meet weekly to discuss administrative support for engagement.

#### Support for Patient and Other Stakeholder Engagement Through Organizational Processes

Our initial orientation of stakeholders to the COMPASS Study includes a discussion of the overall importance of engaging stakeholders in research, and the various levels of and opportunities for engagement (and compensation) in COMPASS, in particular. Our goal is not to turn stakeholders into researchers but to allow their expertise to shape the study design, implementation, and dissemination. We clarify that we are seeking partners with complementary strengths and unique perspectives on the health-care system and that understanding their needs is critical to the success of the project.

During the pre-award period, the researchers developed a stakeholder management plan (“roadmap”) outlining where stakeholders could influence the study. The study team firmly believed that engagement had to be an iterative process in the post-funding phase because, if we truly partnered with stakeholders, their influence could certainly influence the study direction. Creating a stakeholder roadmap allowed us to target defined actions to engage stakeholders quickly, even before the study begun. Our stakeholder engagement plan focused on 4 key sets of activities: (1) study planning (including study design, intervention design and procedures, outcomes measurement, materials); (2) hospital/patient recruitment and retention; (3) study implementation; and (4) translation, including interpreting study findings and disseminating results back to participating communities and the public. Within each activity, we have a series of circumscribed projects defined (see Table 2).

**Table 2:** The Comprehensive Post-Acute Stroke Service (COMPASS) study stakeholder engagement roadmap

Period of engagement	Activities
Study oversight	Participate in Steering Committee meetings to be part of decision making Participate in subcommittees/working groups to have input throughout the study
Intervention	Design intervention components Caregiver support Recovery and physical activity for stroke survivor Secondary prevention and medication management Community resources for stroke survivors and caregivers State of stroke care at participating hospitals (transitional care survey)
	Develop messaging and marketing for patients, caregivers, and providers
	Define goals/screener questions for 2-d call to stroke survivors in intervention arm
	Define goals/measures/tool kits for 7–14-d clinic/home visit for stroke survivors in intervention arm Functional assessment Medical and neurological assessment Electronic care plan and database Referrals of other providers Caregiver assessment
	Define goals for 30-d and 60-d follow-up calls to stroke survivors in intervention arm
	Identify community resources to support stroke survivor recovery after discharge
	Network across communities to identify their constituents, visit communities as we develop community coalitions
	Help develop training of hospital-based and community-based clinicians participating in the intervention
	Draft job description for post-acute care coordinator
	Provide input on study Web site—input on Web site content, user friendliness
Outcome measurement	Finalize 90-d patient outcome measurement
	Finalize 95–110-d caregiver outcome measurement
	Refine data collection forms (including enrollment form)
	Refine telephone scripts used with patient at data collection
	Provide input on consent process and wording of consent forms
	Help optimize methods of data collection, brainstorm on how to address nonresponse of participants
	Help design best ways to engage patients/caregivers between discharge and 90-d data collection
	Give input on claim-based outcome measures (mortality, hospitalization, physician visits, medication use, etc.)
QI	Guide development and reporting of the QI metrics most meaningful for providers and health-care systems
Recruitment and retention of hospitals and patients	Help design a patient-facing informational brochure about the study
	Provide input on how best to incentivize partner hospitals to follow patients and collect relevant data in a timely manner, and to implement full intervention
	Help determine how best to identify and follow patients and determine study-eligible patients
	Give input on how best to keep hospital/staff engaged in study
	Advise on methods for monitoring and maintaining completeness of patient enrollment
Data analysis	Formulate secondary questions that matter to stakeholders
Dissemination	Refine dissemination and implementation plan
	Leverage stakeholders’ networks to maximize reach
	Disseminate study information and final results across the state
	Identify barriers for dissemination
	Identify most effective dissemination strategies to ensure timely and effective communication to patients and caregivers, community leaders, hospital administrators, policy makers
	Educate local (eg, county commissioners, mayors) and state policy makers (eg, state senators) to understand the kind of research we are doing, why it is important, why North Carolina is uniquely positioned to do this and become a national leader, to understand barriers to providers and patients

QI, quality improvement.

Given the rapid start-up phase, we wanted to be sure to capture any additional opportunities for engagement. Thus, we have designated and trained 1 researcher in each study subgroup to be the engagement advocate. This person notifies the faculty engagement officer either when engagement has occurred or when additional engagement of stakeholders will be needed as the study progresses. This information is tracked in the Research Electronic Data CAPture (REDCap) data system’s engagement tracker, which is described below.

#### Communication with Patients and Other Stakeholders

For stakeholders not continuously engaged in the COMPASS Study in the ways already noted, study updates are provided via conference calls, or by COMPASS team member updates at regional meetings that those stakeholders were already scheduled to attend. The team includes skilled facilitators with expertise in leading focus groups or group feedback sessions. In addition, the team includes experts in primary care practice and pharmacy quality improvement; physical therapy; and family caregiver needs. They engage with their respective communities for this study where their contacts and stature as opinion leaders are most effective. An important component of our stakeholder roadmap is explaining to partners how their feedback was incorporated and, if not, why not—and in a timely manner.

Face-to-face contact is invaluable for building relationships, and all study team members have spent significant time traveling to meet with stakeholders. Yet, limited time and resources for state-wide travel necessitate using regular conference calls, webinars, and email contacts. When patient and caregiver stakeholders do not have email access or cell phone minutes, we call them at their homes. Openness to special arrangements is important. For example, to connect with 1 highly engaged couple (each both a stroke survivor and a caregiver) in rural NC, we email their neighbor. This person prints out emails and drives them to our partners, who then call us from their house phone when they have time to talk. We also mail documents to our partners when that seems most practical.

#### Transparent Involvement Processes

Including patients, family caregivers, clinicians, and organizations dedicated to improving care for stroke patients in COMPASS subgroups, including the Steering Committee, allows for information exchange, keeps the patient perspective central, and fosters transparency. To make our engagement processes clear, we clarify roles, expectations, compensation, workload, timelines, deliverables, and decision-making processes.

Most of our stakeholder interactions are defined by the IRB as non-research activities (eg, committee work, team meetings), but some are defined as research and require IRB approval (eg, interviews, focus groups). This distinction is made explicit to our stakeholders.

Our written compensation policy has evolved to account for stakeholder feedback while meeting PCORI principles of reciprocal relationships, trust and transparency, and budget constraints. For example, we do not reimburse clinical or industry partners for their participation, but we do reimburse patient and caregiver participants. Because employees of our main community-based partner cannot accept consulting fees, we budgeted support to the organization in a subcontract. We make clear in advance when we cannot compensate individuals for their time in an upcoming activity. Some choose to participate and others decline. This flexible participation is an integral part of our model.

#### Tracking of Engagement

**Fig. 2:**
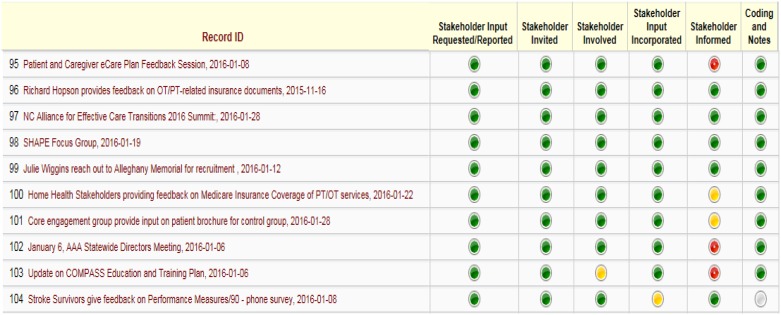
Screenshots from the Research Electronic Data CAPture engagement tracker.

Central to our infrastructure is a system to capture engagement work and stakeholder feedback so that we can track what actions were ultimately taken based on their input and to better delineate the impact of a given strategy on outcomes. To do this, we developed an engagement database within the REDCap software system, a secure platform for research data collection and analyses developed within the Clinical and Translational Science Award network [21]. Our database was designed to (1) document all engagement activities systematically, (2) monitor the complexities of our engagement process, (3) facilitate data collection for the annual engagement report to PCORI, and (4) tie engagement activities to processes and eventual outcomes. Data include which stakeholder groups were involved; when, where, why, and how they were engaged; their level of engagement (using PCORI’s framework); how challenges to engagement were overcome, and how stakeholder feedback shaped the study. The database includes a dashboard to visually track each engagement activity and help the engagement team ensure that activities are on schedule (see Fig. 2).

#### Evaluation of Engagement

We will monitor our engagement process through proactive oversight of milestones and deliverables spelled out in our PCORI contract and engagement proposal.

We will perform quarterly check-ins via survey to ask all patient and stakeholder partners if they feel their perspectives are valued by the study team, if they are satisfied with their level of involvement, if they are involved in the activities they want to be involved in, and if the methods used to elicit their feedback match their preferred style of communication. These data will be used to continuously improve study processes, which signals to all stakeholders the power and value of their voice.

## Conclusion

This paper describes the comprehensive methods used to incorporate stakeholders in the planning and design of a large pragmatic trial. To date, we have successfully included perspectives and input from multiple stakeholder groups in a timely fashion because our efforts were driven by a sound conceptual model and built on an existing state-wide infrastructure. This paper is intended to provide guidance to others planning or conducting stakeholder-engaged research as we collectively attempt to identify evidence-based approaches by which engagement can enhance research and close the gap from evidence to action.

## Supplementary material

For supplementary material accompanying this paper visit https://doi.org/10.1017/cts.2016.26. click here to view supplementary material
