# Supplementary material for: Methods guiding stakeholder engagement in planning a pragmatic study on changing stroke systems of care
Source: J Clin Transl Sci. 2017 Feb 27;1(2):121–8. doi: 10.1017/cts.2016.26 (PMC5471818; doi:10.1017/cts.2016.26)
Supplement: Supplementary file 1 [file S2059866116000261sup001.pdf]

COMprehensive Post-Acute Stroke Services (COMPASS) Study

Principal-Investigator:

- Pamela Duncan, PhD, PT

Co-Investigators:
Wake Forest

- Cheryl Bushnell, MD, MHS (Co-PI)
- Walter Ambrosius, PhD
- Ralph D`Agostino, PhD
- Sabina Gesell, PhD
- Allison Brashear, MD, MBA
- Brian Wells, MD, PhD

UNC Chapel Hill

- Wayne Rosamond, PhD (Co-PI)
- Janet Freburger, PhD, MS
- Jacqueline Halladay, MD, MPH
- Anna Kucharska-Newton, PhD, MPH, MS

UNC Wilmington

- Barbara Lutz, PhD, RN

East Carolina University

- Doyle "Skip" Cummings, PharmD

Duke University

- Janet Prvu Bettger, ScD
- Amy Pastva, PhD, PT

Sponsor:
Patient-Centered Outcomes Research Institute (PCORI)

Contents

COMprehensive Post-Acute Stroke Services (COMPASS) Study 1
Background 4
Objectives & Specific Aims 5
Primary Study Question: 5
Primary Aim: 5
Secondary Aims: 5
Exploratory Aims: 5
Methods and Measures 6
Study Design 6
Study Setting 6
Study Population 6
Randomization 6
Subjects Selection Criteria 7
Inclusion Criteria: 7
Exclusion Criteria: 7
Sample Size: 7
Intervention and Interactions 8
Patients in Control Hospitals 8
Patients in COMPASS hospitals 9
Caregivers 11
Community-Engagement 11
Outcome Measures 12
Patient Participant Study Outcomes: 12
Caregiver Participant Study Outcomes: 14
Study outcomes collected and linked to insurance claims data: 15
Clinical Data collection: 16
Analytical Plan 17
Human Subjects Protection 19
Informed Consent 21
Confidentiality and Privacy 23
Overview 23
COMPASS Analytic Database at EMSPIC 23
Electronic Care (eCare) Plan Informatics Database 24
Carolina Survey Research Lab Database and Security 24
Sheps Center Data Security 25
Description of the Secure Data Transfers 27

Data Access for Analysis 28
REDCap (Research Electronic Data Capture) 28
Data and Safety Monitoring 30
Responsibilities 30
Frequency of Meetings and Communication between DSMB and COMPASS 30
Membership 31
Reporting of Unanticipated Problems, Adverse Events or Deviations 31
References 32
Appendices 34

Background

Stroke is the fifth-leading cause of death in the United States (US) and a major cause of long-term disability.[1] North Carolina (NC) is in the Stroke Belt, a region of the US with a very high stroke incidence. Eastern NC is the “buckle” of the Stroke Belt, where stroke mortality is 40% higher than the US average and hospital admission rates are the highest in NC.[2] African Americans, over 20% of the population in NC, are more likely than their white counterparts to die of stroke at relatively young ages.[2] There is also evidence that African-Americans are more likely to be readmitted after stroke.[3]

Stroke exemplifies a complex co-morbidity condition, with 85% of Medicare beneficiaries with stroke having four or more other chronic health conditions,[4] and their health care is costly. Stroke patients with congestive heart failure have per capita costs that are about five times higher than the average spending for Medicare fee-for-service beneficiaries.[4] In addition, stroke patients are frequently readmitted to the hospital.[5,6] A PCORI-funded study shows that 25% of stroke patients discharged home without post-acute care are readmitted within 90 days[7]. These data align with what stroke survivors and caregiver advocates from across NC have said that stroke patients need. One of our patient partners who started a support group for other stroke survivors stated:
“You can’t just place an individual back in their home with a bottle of pills and a follow-up visit…There is a real need for assistance when patients get home…What is in place for the patient? Nothing… No visiting nurse, no one to answer questions, or help them get what they need. That is why people end up back in the hospital.”

Roughly half of stroke patients in NC are discharged directly home, often with new disability. Around 44% cannot walk independently at discharge.[8] Complications from immobility account for up to 51% of deaths in the first 30 days after ischemic stroke.[9,10] Other complications are common early after stroke and include falls and fractures, aspiration pneumonia, deep vein thrombosis, infections, depression, and adverse events associated with warfarin therapy.[11–16] Even those with mild post-stroke disability can have physical and cognitive deficits which often go undetected during acute hospitalization. These mild disabilities interfere with function, and management of risk factors and medication.[17,18]

Fewer than 50% of individuals with stroke have their risk factors assessed, treated, or controlled. Of those overweight at initial evaluation, 90% remain overweight. Nearly half of hypertensive individuals do not have blood pressure controlled. Smokers do not quit and few participate in exercise programs.[19] At three months post-discharge, only 75% of stroke patients are still taking their preventive medications.[20] About 40% remain dependent on others.[20,21] Stroke also affects caregivers. Caregivers have poorer mental health, less social contact and activity, and are at increased risk for depression.[20,21]

Comprehensive post-acute services for stroke require bridging hospital-based acute care with expanded care teams for rehabilitation, primary care management, access to community resources, and caregiver support. Evidence-based reviews have concluded that stroke morbidity and mortality could be reduced through effective transitional care,[22–24] secondary prevention,[25] and rehabilitation early post-stroke.[26] Involvement with a stroke liaison worker or case manager is associated with more knowledge about stroke and satisfaction with services.[27,28] Caregiver-oriented, individualized discharge planning for stroke patients going home improves caregiver preparedness.[29]

Given the significant impact of stroke on public health, the high risk and complexity of these patients early after discharge, and the strain on caregivers, an effective post-acute care model is needed. A pragmatic trial is the ideal method to test a care model that can be readily disseminated and implemented.

Objectives & Specific Aims

The COMprehensive Post-Acute Stroke Services (COMPASS) Study will determine the effectiveness of a post-acute comprehensive intervention.

Primary Study Question:

Does implementation of the COMPASS for stroke patients discharged directly home, improve functional outcomes (measured by the Stroke Impact Scale-16) at 90 days post-stroke? Intention-to-treat principles will be used to determine all primary and secondary outcomes.

Primary Aim:

Comparative effectiveness of COMPASS verses usual care (Control) on stroke survivor functional status at 90 days post-stroke.

Secondary Aims:

1) Using responses from the 90 day caregiver survey, determine if the COMPASS intervention reduces caregiver strain (measured by the Modified Caregiver Strain Index) at 90 days post-stroke.
2) Using claims data from multiple payer sources up to 12 months after stroke hospitalization, we will measure effectiveness of COMPASS vs usual care for all-cause readmissions at 30 and 90 days post-discharge.
3) Using responses from the 90 day survey, we will measure general health; global disability; physical activity; depression (PHQ2); cognition (Mini MOCA); medication adherence (MMAS-4); management of blood pressure; falls; fatigue; satisfaction of care; and use of community services.
4) Comparative effectiveness of the COMPASS vs usual care on: mortality; health care utilization; (emergency department visits, hospitalizations, admissions to skilled nursing facilities/inpatient rehabilitation facilities); and use of transitional care management billing codes. We will use claims data from multiple payer sources up to 12 months after stroke hospitalization.
5) The effectiveness of the COMPASS intervention on primary and secondary outcomes by race, sex, age, stroke severity, and insurance status.

Exploratory Aims:

1) Evaluate compliance with the new model of post-acute stroke care by exploring quality indicators among intervention hospitals, (e.g., proportion of patients called within 2 days after hospital discharge; proportion of patients seen by an advanced practice provider [MD/NP/PA] within 7 to 14 days from hospital discharge; and proportion of eligible patients receiving home or outpatient rehabilitation therapy).
2) Compare Phase 1 and Phase 2 performance indicator reporting and outcomes from claims data.

Methods and Measures

Study Design

The COMPASS Study is a pragmatic, cluster-randomized trial of 50 hospitals in North Carolina designed to determine the effectiveness of a model of post-acute stroke care (i.e. the COMPASS Intervention) compared with usual care (Control).

Study Setting

The COMPASS intervention will be implemented in hospitals and communities in 2 phases over a 5-year period. We will recruit 50 hospitals that represent diverse geographic locations (i.e. rural vs urban), primary stroke center certification status, and stroke patient volumes. Included in this IRB Application (Appendix 1) is an attachment (Titled: COMPASS Hospital Characteristics) which provides a side-by-side comparison of hospitals expected to participate in the COMPASS trial with All North Carolina Hospitals. A list of our anticipated COMPASS Hospitals is also included. The data used to make the comparison is from CMS Hospital Compare[30]. COMPASS will ask participating hospitals to fill out a Hospital Survey (Appendix 2) at the start of the study to better understand the current state of transitional care at each hospital.

Study Population

In 2013, data from hospitals in the North Carolina Stroke Care Collaborative (NCSCC) indicated that 46% of patients were discharged directly home from the hospital after a stroke (our proposed study population). Using this data we anticipate an estimated sample of approximately 6,000 potentially eligible participants. In that population, the mean age was 65.0 years (SD 14.4), 25% were African American, and 48% were women. Stroke severity, measured by the NIH Stroke Severity score and ranging from 0 (no deficit) to 42 (maximum deficits), was on average 3.2 for those discharged home.

Randomization

Since individual stroke patients cannot easily be randomized to receive the COMPASS intervention, we determined that the optimal statistical design for this pragmatic trial utilizes a cluster randomized approach. Thus, 50 individual hospitals will be randomized to either receive the COMPASS intervention at the beginning of the study (Phase 1) or in Phase 2 (see Figure 1). Hospitals randomized to receive the intervention in Phase 2 will be referred to as the control group. Randomization per hospital will be stratified by volume of stroke patients and primary stroke center status. In this intention-to-treat design, all stroke patients who are discharged directly home from randomized acute care hospitals will be included in analyses. The analyses will be performed at the individual level with adjustment for lack of independence between hospitals. The primary analysis of patient-centered outcomes will occur at the end of Phase 1.

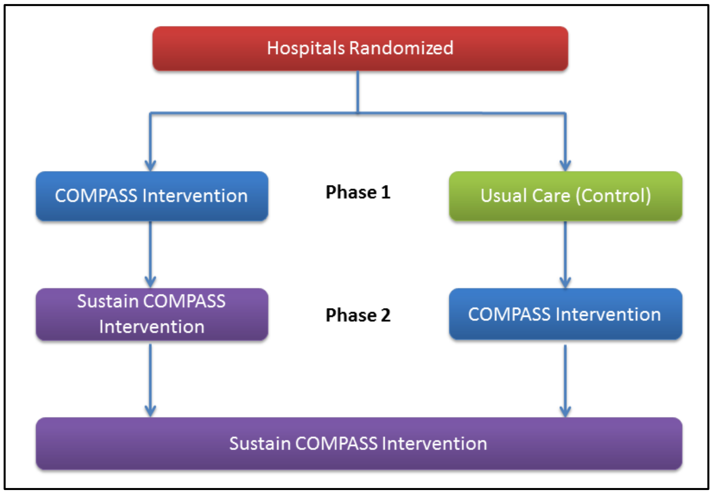

Figure 1: Hospital Randomization to the COMPASS Intervention in Phase 1 and Phase 2

Subjects Selection Criteria

Inclusion Criteria:

- Patients aged 18 years or older, discharged home from a participating COMPASS Study hospital with a diagnosis of ischemic or hemorrhagic stroke or transient ischemic attack (TIA).

Exclusion Criteria:

- Patients transferred to another short-term acute care hospital, skilled nursing facility, inpatient rehabilitation facility, or hospice.
- Patients with a diagnosis of subdural or aneurysmal subarachnoid hemorrhage.
- Patients who speak neither English nor Spanish.

Sample Size:

- Approximately 6,000 potentially eligible participants per year.

Intervention and Interactions

COMPASS will support hospitals in implementing structured post-acute stroke services consistent with CMS transitional care codes and management. Hospitals which participate are implementing COMPASS as the new standard of care. The goal of COMPASS is to structure and organize the processes of post-acute care to optimize patient recovery function, reduce caregiver stress, improve risk factor management, and facilitate self-management of risk factors.

The Intervention has two study arms. Figure 2 depicts the patient flow of activities for the control and COMPASS.

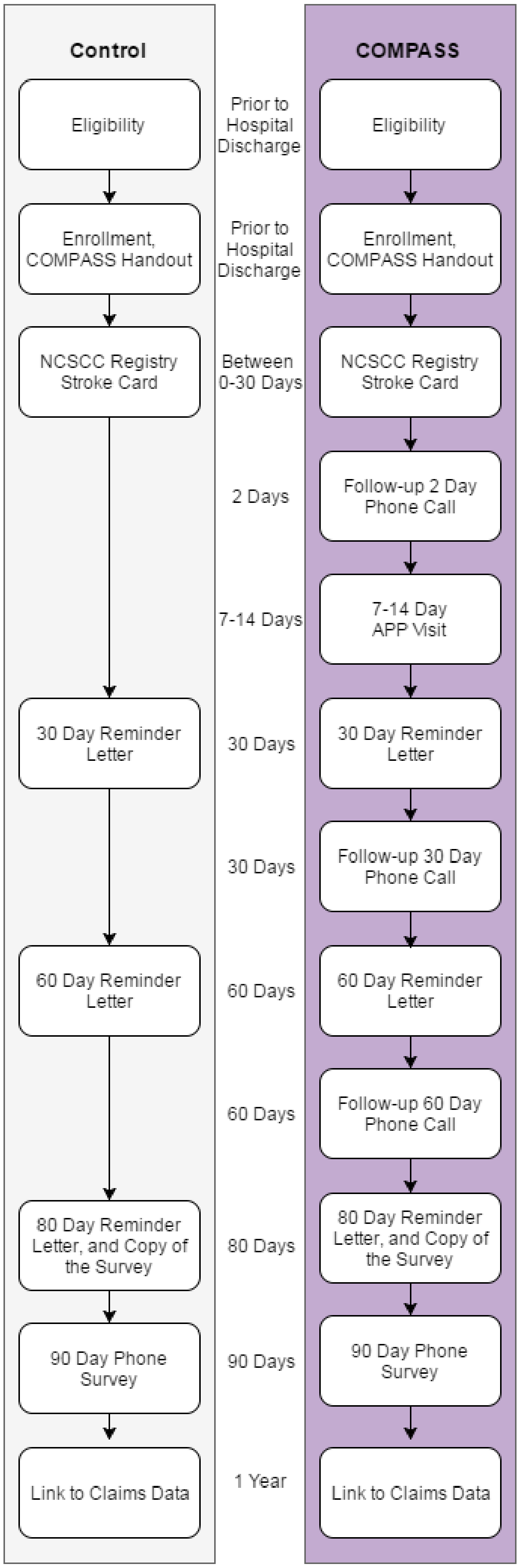

Figure 2: Flow of Intervention Activities for Control and COMPASS Participants

Patients in Control Hospitals

Prior to hospital discharge, the Post-Acute Coordinator (PAC) will identify stroke and TIA patients for eligibility using the Eligibility Screening Form (Appendix 3). Determining eligibly will involve daily review of stroke admissions to the hospital by screening the electronic medical records. This will be done under HIPAA Waiver.

If eligible, the patient will be enrolled, a COMPASS ID is assigned and the PAC will fill out the Enrollment Form (Appendix 4). For those not eligible, no further information is collected and a COMPASS Identification number is not assigned.

The PAC will visit eligible patients in the hospital, notify the patient that the hospital is participating in the COMPASS Study, and give the patient a handout with information on the COMPASS Study (Appendix 5). The information informs the patient that their hospital is participating in a state-wide study to evaluate best models of post stroke care. The patient will be informed if their hospital is in the usual care group of in the COMPASS intervention group. The handout that patients receive in the control arm is tailored to the hospital. Each control arm hospital brochure will include the PAC name, the PAC contact information and a tailored description of the post-acute "standard care" that the patient will receive at that hospital. This information will equip patients with information to fully inform them of the COMPASS Study and how this will impact them and what to expect from the hospital. The PAC will also explain that the patient will get a phone call in about three months asking them to participate in a telephone survey and that they will get three reminder letters in the mail to remind them about the survey (Appendix 6-8). The PAC will record patient and caregiver contact information on the COMPASS enrollment form. In the COMPASS analytical database, the PAC will record the date, time and method (i.e. in person or over the phone) of informing the patient of the

COMPASS Study, and confirm that the patient was given the brochure. As a pragmatic study, we anticipate that PACs may miss a patient (for example a patient may be missed on the weekends) and the patient will need to be notified of the study over the phone and mailed the brochure.

As part of case ascertainment and consistent with the North Carolina Stroke Care Collaborative (NCSCC) methodologies to characterize all stroke admissions and processes of acute stroke care the PAC will complete the NCSCC Stroke Care Card (Appendix 9).

At 90 Days, stroke survivors will receive a phone call from the Carolina Survey Research Lab (CSRL). CSRL will follow a telephone script which includes consent (Appendix 10) and conduct blinded assessments (Appendix 23 and 24). If the patient prefers s/he can request that a proxy answer questions and provide verbal consent and HIPAA Authorization on her/his behalf. During this 90 survey we will ask the patient for permission to contact the primary caregiver to complete a caregiver assessment.

Approximately one year later the study will acquire claims data from Medicare, Medicaid, State Insurance Plan, and Blue Cross Blue Shield. We plan to use a HIPAA Waiver to acquire the data sets and along with this application we are asking for a waiver of signed consent to link all enrolled patients to the claims data sets.

Patients in COMPASS hospitals

In addition to the activities outlined for Control Hospitals, patients who enter the study through a COMPASS Intervention hospital will receive: (1) a follow-up phone call 2 days after being discharged from the hospital, (2) a 7-14 day Advanced Practice Provider visit, (3) a follow-up 30 day phone call, and (4) a follow-up 60 day phone call. The structure and processes of this COMPASS intervention are consistent with the Center for Medicare and Medicaid (CMS) Transitional Care Management Codes. In essence the COMPASS study is an evaluation of the implementation of CMS recommendations for post hospital care coordination.

The process for determining Eligibility for COMPASS patients is identical to that of Control patients.

If the patient is eligible, they will be enrolled in a similar manner to that of the Control patients. The PAC will visit each patient in the hospital and give the patient a tailored handout about the COMPASS Study (Appendix 12). The information informs the patient that their hospital is participating in a state-wide study to evaluate best models of post stroke care. The patient will be informed if their hospital is in the usual care group of in the COMPASS intervention group. This brochure is tailored to the hospital. The PAC name and the PAC contact information will be provided on an appointment card. This information will equip patients with information to fully inform them of the COMPASS Study and how this will impact them and what to expect from the hospital. The PAC will also give an additional handout about COMPASS intervention activities (Appendix 13), a Blood Pressure Log (Appendix 14) and Blood Pressure Handout (Appendix 15). The PAC will work with the patient to schedule follow-up visits with the patients Primary Care Physician (PCP) and the Advanced Practice Provider (APP) for the 7-14 day follow-up visit. The PAC will let the patient know they will be calling them in a couple of days for the 2-day follow-up phone call. The PAC will record patient and caregiver contact information on the COMPASS enrollment form. In the COMPASS analytical database, the PAC will record the date, time and method (i.e. in person or over the phone) of informing the patient of the COMPASS Study, and confirm that the patient was given the brochure. As a pragmatic study, we anticipate that PACs may miss a patient (for example a patient may be missed on the weekends) and the patient will need to be notified of the study over the phone and mailed the brochure.

As part of case ascertainment and consistent with the North Carolina Stroke Care Collaborative methodologies to characterize all stroke admissions and processes of acute stroke care the PAC will complete the NC Stroke Care Card (Appendix 9). **(Note: this is identical to the Control group**).

At 2 days post- hospital discharge, the PAC will call the patient and discuss medication use, symptoms, and confirm (or schedule) follow-up appointments with the patients Primary Care Physician and the Advanced Practice Provider. The PAC will provide patient education to ensure they know about signs of a subsequent stroke. The script for the 2 day follow-up call is in Appendix 16.

Between 7 and 14 days, the patient will attend a follow-up visit with the advanced practice provider (APP). The PAC will also attend this visit. The goal of this visit is to create an individualized patient Care Plan and, if needed, make additional referrals for the patient. At this visit, the PAC will clinically assess the patient using the Post-stroke Functional Assessment (Appendix 17). Based on responses from this assessment, the PAC may also conduct the Caregiver Assessment (Appendix 18) with the patient's caregiver if the caregiver is present. The provider will assess the patient using the Post-stroke Advanced Practice Assessment (Appendix 19). Responses to these assessments will be used by the provider to develop an individualized patient Care Plan. These three assessments have been programmed into an electronic platform (eCare Application) to ease administration, assessments, data capture and development of the individualized patient Care Plan (eCare Plan) which will be conducted on an iPad. The tool will summarize the three assessments and make suggestions for the provider in creation of the individualized patient Care Plan (Appendix 20 is an example; the provider customizes these recommendations). As the decision-making authority in patient care, the final individualized patient Care Plan and any referrals are given by the provider. The PAC will review the Care Plan with the patient, establish preferences for care and coordinate referrals for services. A copy of the Care Plan will go forward to the patient’s primary care provider and rehabilitation providers. The APP will send a summary of the visit and the Care Plan to the patient’s primary care provider and rehabilitation providers. At the completion of the visit, the patient will be asked for consent and HIPAA Authorization to use clinical data for future analyses (Appendix 21).

At 30 and 60 days, patients will be called by the PAC to follow-up on their Care Plan. The PAC will ask the patient if they are having any challenges with implementing the care and treatment plans that their health providers have given them (Appendix 22).

Patients will also receive three letters in the mail to remind them about the 90 day survey and to provide educational information and resources from the American Stroke Association (Appendix 6-8). Although the 90 day phone survey will be relying on a Full HIPAA Authorization, the letter mailed to the patient at 80 days includes a statement on HIPAA to inform the patient on how they have been identified for the study and that agreeing to participate in the phone survey will be authorizing the use of identifiable health information and how that information will be used in the study. **(Note: this is identical to the Control group**).

At 90 Days, stroke survivors will receive a phone call from the Carolina Survey Research Lab (CSRL). CSRL will follow a telephone script which includes consent (Appendix 10) and conduct blinded assessments (Appendix 23 and 24). If the patient prefers s/he can request that a proxy answer questions and provide verbal consent and HIPAA Authorization on her/his behalf. During this 90 survey we will ask the patient for permission to contact the primary caregiver to complete a caregiver assessment.

Approximately one year later the study will acquire claims data from Medicare, Medicaid, State Insurance Plan, and Blue Cross Blue Shield. We plan to use a HIPAA Waiver to acquire the data sets and along

with this application we are asking for a waiver of signed consent to link all enrolled patients to the claims data sets. **(Note: this is identical to the Control group**).

Caregivers

As described above, patients will be asked on the 90 day phone survey for permission to send a paper survey to their caregiver. If the patient permits, the patient will provide the primary caregiver information and the COMPASS Study team will mail the caregiver a letter (Appendix 26), the Caregiver Survey (Appendix 27) and the Proxy SIS (Appendix 28). Non-respondents will be mailed a second letter (Appendix 29) with surveys (Appendix 27-28). If the caregiver still does not respond they will receive a reminder telephone call from UNC Carolina Survey Research Lab. Once the caregiver participant completes the survey, the study team will send a $10 Visa gift card with a short thank you note (Appendix 30).

Community-Engagement

This is a community-engaged study. Patients, family caregivers, and others stakeholders will be involved in all phases of the research process. These community members will be engaged in non-research activities (e.g., revising study materials for clarity) as well as research activities (e.g., participating in focus groups). By design, community-engaged research requires decision making by many stakeholders and frequent IRB amendments. For clarity and oversight purposes we have submitted a separate IRB (PCORI Stakeholder Interviews: IRB00028495; Appendix 31) for research activities involving stakeholders.

Outcome Measures

Patient Participant Study Outcomes:

Measure	Assessment(s)	When Collected	Appendix
**Physical Function**	Stroke Impact Scale (SIS-16)	@90 days, consenting patients asked by CSRL	23
**Physical Function**	Proxy Stroke Impact Scale (Proxy SIS-16)	@95 days, mailed to the caregiver.	28
**Self-rated General Health**	A question rating health on a 5-point scale & a question on perception of health improvement	@90 days, consenting patients asked by CSRL	24
**Disability and Dependence**	Modified Rankin Scale	@90 days, consenting patients asked by CSRL	24
**Physical Activity**	Three questions which ask about time spent walking	@90 days, consenting patients asked by CSRL	24
**Depression**	Patient Health Questionnaire (PHQ-2) Note: these items do not ask about suicidal thoughts or actions	@90 days, consenting patients asked by CSRL	24
**Cognition**	MOntreal Cognitive Assessment (MOCA) Mini	@90 days, consenting patients asked by CSRL	24
**Medication Adherence**	Morisky Medication Adherence Scale (MMAS-4)	@90 days, consenting patients asked by CSRL	24
**Secondary Prevention Self-Management**	By asking: "Do you check your blood pressure at home?"	@90 days, consenting patients asked by CSRL	24
**Blood Pressure Management Effectiveness**	For those who check BP at home, we ask: "Is your blood pressure less than 140/90 most of the time?"	@90 days, consenting patients asked by CSRL	24
**Falls and Hospitalization**	Questions to capture: Number of falls, injuries, and hospitalizations	@90 days, consenting patients asked by CSRL	24
**Fatigue**	PROMIS Fatigue Instrument – Adult Short Form 4A	@90 days, consenting patients asked by CSRL	24
**Satisfaction with care**	Questions on how the patient felt about care and treatment from health care providers.	@90 days, consenting patients asked by CSRL	24
**Use of Community Resources**	By asking: "Since discharge from the hospital, have you used services such as Senior	@90 days, consenting patients asked by CSRL	24

	Services, Meals on Wheels, in-home aides, or stroke survivor or caregiver support groups?”		
**Initial Presentation Data**	Hospital arrival and Mode of arrival, Ambulatory status prior to admission, Diagnosis at admission, NIHSS, Imaging performed, etc.	Entered into the COMPASS Database by the hospital	9
**Demographic Data**	DOB, Race, Gender, Insurance, Medical History, Medication , etc.	Entered into the COMPASS Database by the hospital	9
**t-PA Data**	Time t-PA was initiated, BP and Glucose levels, bleeding complications, etc.	Entered into the COMPASS Database by the hospital	9
**In-hospital Data**	Admission data, secondary prevention counseling, treatment, lipid profile, medications, treatments, etc.	Entered into the COMPASS Database by the hospital	9
**Discharge Data**	Resources and stroke education materials, assess for rehabilitation, ambulatory status, Rankin Score, final diagnosis, discharge disposition, ICD-10 data, etc.	Entered into the COMPASS Database by the hospital	9

Caregiver Participant Study Outcomes:

Measure	Assessment(s)	When Collected	Appendix
**Caregiver Burden**	Modified Caregiver Strain Index	@95 days, mailed to the caregiver	27
**Relation to stroke patient**	Relation to stroke patient	@95 days, mailed to the caregiver	27
**Demographics**	Age, Gender, Race	@95 days, mailed to the caregiver	27
**Primary Caregiver**	Are you the primary caregiver	@95 days, mailed to the caregiver	27
**Length of Caregiving service**	How long have you been providing care?<br>How many hours per day do you spend providing care?<br>Do others help provide care?	@95 days, mailed to the caregiver	27
**Type of caregiving activities**	Type of caregiving activities	@95 days, mailed to the caregiver	27
**Awareness and use of Community Resources**	Awareness and use of Community Resources	@95 days, mailed to the caregiver	27
**Self-rated General Health**	Compared to others your age, how would you rate your health using a scale of 1 to 5, with 1 being “Poor” and 5 being “Excellent?”	@95 days, mailed to the caregiver	27
**Accessed the COMPASS Website**	Have you explored the information on the COMPASS website	@95 days, mailed to the caregiver	27

Study outcomes collected and linked to insurance claims data:

Measure	Assessment(s)	When Collected	Appendix
**Readmissions**	30-day and 90-day all-cause readmission	Approximately 1 year post-stroke	This will be collected via Administrative Claims Data sets from Medicare, Medicaid, State Insurance Plan, and Blue Cross Blue Shield
**Mortality**	Mortality	Approximately 1 year post-stroke	This will be collected via Administrative Claims Data sets from Medicare, Medicaid, State Insurance Plan, and Blue Cross Blue Shield
**Emergency Department (ED) Visits**	Number of patient emergency department visits	Approximately 1 year post-stroke	This will be collected via Administrative Claims Data sets from Medicare, Medicaid, State Insurance Plan, and Blue Cross Blue Shield
**Hospitalizations**	Number of patient hospitalizations and number of hospital days	Approximately 1 year post-stroke	This will be collected via Administrative Claims Data sets from Medicare, Medicaid, State Insurance Plan, and Blue Cross Blue Shield
**Admissions to skilled nursing facilities and inpatient rehabilitation facilities**	Number of patient admissions and number of days in to skilled nursing facilities, and number of patient admissions to inpatient rehabilitation facilities	Approximately 1 year post-stroke	This will be collected via Administrative Claims Data sets from Medicare, Medicaid, State Insurance Plan, and Blue Cross Blue Shield
**Use of transitional billing codes**	Use of Transitional Care Management (TCM) billing codes and Chronic Care Management (CCM) billing codes	Approximately 1 year post-stroke	This will be collected via Administrative Claims Data sets from Medicare, Medicaid, State Insurance Plan, and Blue Cross Blue Shield

Clinical Data collection:

- We will collect clinical data to inform the patient's individualized care plan that will be routine for implementing transitional care, and would like to keep this data for future analyses:

Measure	Assessment(s)	When Collected	Appendix
**Neurological Status and Deficits**	Post Stroke Advanced Practice Assessment	7-14 Day Visit	19
**Stroke Complications**	Post Stroke Advanced Practice Assessment	7-14 Day Visit	19
**Stroke Risk Factor Management**	Post Stroke Advanced Practice Assessment	7-14 Day Visit	19
**Lifestyle Coaching**	Post Stroke Advanced Practice Assessment	7-14 Day Visit	19
**Medication Access and Use**	Post Stroke Functional Assessment	7-14 Day Visit	17
**Knowledge of Stroke Risk Factors**	Post Stroke Functional Assessment	7-14 Day Visit	17
**Self-rated General Health**	Post Stroke Functional Assessment	7-14 Day Visit	17
**Mobility,**	Post Stroke Functional Assessment	7-14 Day Visit	17
**Falls and Hospitalizations**	Post Stroke Functional Assessment	7-14 Day Visit	17
**Social and Caregiver Support**	Post Stroke Functional Assessment	7-14 Day Visit	17
**Activities of Daily Living**	Post Stroke Functional Assessment	7-14 Day Visit	17
**Home Health, Outpatient services**	Post Stroke Functional Assessment	7-14 Day Visit	17
**Durable Medical Equipment**	Post Stroke Functional Assessment	7-14 Day Visit	17
**Living Will**	Post Stroke Functional Assessment	7-14 Day Visit	17
**Relation**	Caregiver Assessment	7-14 Day Visit	18
**Demographics**	Caregiver Assessment	7-14 Day Visit	18
**Caregiving activities**	Caregiver Assessment	7-14 Day Visit	18
**Self-rated General Health**	Caregiver Assessment	7-14 Day Visit	18
**Stress**	Caregiver Assessment	7-14 Day Visit	18
**Signs of a stroke**	Caregiver Assessment	7-14 Day Visit	18

Analytical Plan

As described above, this pragmatic trial utilizes a cluster randomized design with 50 hospitals being randomized to receive the COMPASS intervention (N=25) or control (N=25) in Phase 1. In Phase 2, the control group hospitals will be rolled into the intervention (Figure 1). All stroke patients who are discharged directly home from one of the randomized hospitals will be included in the intention-to-treat analyses. Analyses will be performed at the individual (patient) level, with adjustments for hospital and/or patient level characteristics to control for possible correlations of patients within hospitals.

We used two stratification factors in randomization: annual stroke patient volume per hospital (3 levels: <100, 100-299, 300+ patients) and whether the hospital is a primary stroke center (Yes/No). Thus, there will be a total of 6 strata. We will use a random permuted block design with block size of two; within each stratum we will randomize an even number of hospitals. This will allow us to maintain balance between the treatment groups while also protecting the validity of the randomization process. Study team involved with site selection will not have access to the randomization schedule which will be held by Dr. Walter Ambrosius. Likewise, Dr. Ambrosius will not be involved in site selection. Although patients in the intervention cannot be blinded to their group assignment, interviewers gathering outcome data will be blinded. Our estimated sample size will permit pre-specified subgroup analyses by race, gender, age, stroke severity and insurance status.

For the primary aim, the primary endpoint is the Stroke Impact Scale (SIS-16) measured 90 days post-stroke. The secondary aims include the Modified Caregiver Strain Index at 90 days; 30- and 90-day all-cause readmissions; and mortality, health care utilization, continuity of care, utilization of transitional care, and medication adherence, all measured 1 year after index discharge. In addition, analyses by race, gender, age, stroke severity and insurance status will determine if there is evidence of heterogeneity of the intervention effect across any subgroups. Finally, for the two exploratory aims, we will (1) examine hospital-level measures of stroke care quality indicators in the COMPASS hospitals, and (2) compare administrative claims outcomes and post-acute stroke performance outcomes between COMPASS patients in Phase 1 (intervention phase) and Phase 2 (sustainability phase).

Since the primary endpoint (SIS-16) is measured on a continuous scale, we will use a mixed model to compare the COMPASS and control groups. Although the stratified randomization of hospitals should balance most important hospital-level characteristics between groups, since imbalances may exist between groups on patient-level characteristics, we propose to include both fixed and random effects in this mixed model. The first model will include two fixed effects: stratum (1 to 6) and the intervention effect (COMPASS vs. control) and one random effect: hospital. This additive model can be written as: $Y_{ijk} = \mu + \gamma_k + \alpha_j + \beta_{k(j)} + \varepsilon_{i(jk)}$,where Y_{ijk} is the outcome (i.e. SIS at 90 days) measured on the ith patient, under the jth intervention (j=1 (COMPASS), 2(Control) in the kth hospital; μ is the grand mean; γ_k is the stratum (1 to 6) for hospital k; α_j is the fixed treatment effect for group *j* (COMPASS/CNT); $\beta_{k(j)}$ is the random effect of the kth hospital nested within the exposure group; and $\varepsilon_{i(jk)}$ is the error term for the ith patient nested within the treatment group and hospital. Other fixed effects can be added at the patient level (e.g. age, gender, race, stroke severity, or SES) for sensitivity analyses. The random hospital effect allows the possibility of correlated observations (patients) within hospitals. Of primary interest is the treatment effect (α_j), which indicates difference in the dependent variable (SIS-16) between groups.

After we fit our primary model, we will consider other models that may include more patient-level and hospital-level characteristics. For instance, since some patients may be transferred to a different hospital before being discharged home, we can include a yes/no variable on that point. Although hospitals will be stratified pre-randomization based on stroke volume, we can include a hospital-level covariate for the

total number of stroke patients discharged home for each hospital. With 50 clusters (hospitals) included, we will be able to add other cluster-level covariates to the model if needed.

For secondary aims and outcomes measured on a continuous scale, we will use a similar approach as above (i.e. for the Modified CSI). For binary outcomes such as whether a patient is readmitted within 30 or 90 days (Secondary Aim 2), we will use mixed logit models to fit the relationship between the intervention and outcome measures. The mixed logit model is similar to the mixed model presented above but uses a logit link in a generalized linear mixed model. Software is readily available (e.g., SAS PROC GLIMMIX) that can fit these models. The mixed logit model approach will also allow a mixture of fixed and random effects to be included as in the mixed model above. Other Secondary Aim 2 variables will be analyzed using a mixed model or alternatively generalized linear mixed models (e.g., Poisson regression [with overdispersion] for the number of inpatient days).

We will examine 1-year mortality rates as a binary outcome and use the methods described above to compare groups, but we will also consider mortality as a time-to-event outcome and compare groups using Cox proportional hazards models. In these survival analysis models, the treatment indicator will be included as the primary independent variable and the stratum included as stratification factor.

Human Subjects Protection

COMPASS is implementation of CMS recommendations for post hospital care coordination. COMPASS is using a pragmatic, randomized controlled trial approach because it facilitates consistent delivery in post-acute stroke care management. Hospitals are being asked to implement the COMPASS Intervention as a new standard of care for all stroke patients. Because specific informed consent would not typically be sought in a clinical setting, stroke patients at the participating hospitals will not have the option to consent to (or opt out of) the site intervention. It is unlikely that our study can seamlessly implement the new models of transitional care in routine clinical care delivery if the traditional informed-consent process for research participation is required. COMPASS will incorporate an Integrated Consent Model for Pragmatic Trials.[31] In this model, the "consent" will inform the patient that they are seeking care in a hospital that has been randomized to provide their usual standard of post hospital care or a hospital that is going to incorporate the COMPASS model of post hospital care. This integrated consent model simply incorporates information about the hospital randomization process and whether their hospital is randomized to usual standard of care or the COMPASS intervention.

A stroke coordinator (PAC), who is a hospital employee, will visit patients in the control sites and in the intervention sites prior to hospital discharge and provide patients with a tailored study brochure. The PAC will review the content of the informational brochure (Appendix 5 and Appendix 12) and inform patients that the hospital is participating in a statewide study to evaluate the best way to provide post-acute services after hospitalization for a stroke. The PAC will also inform the patient that there are many ways to care for patients after they leave the hospital and we are not sure which model is best.

The PAC can answer any questions that the patient has regarding this conversation and provide their contact information, the COMPASS toll-free phone number and website as a reference for additional information. For additional assurance, the study will ask PACs to document into the COMPASS Study data portal the date and time the patient was informed. The goal of this study is to capture all patients discharged directly home. In the event that a patient is discharged on a weekend or before the PAC is able to visit the patient, the PAC will have a follow-up phone call with the patient to inform them of the hospital study and then the PAC will mail the brochure to the patient's preferred mailing address. The PAC will also document in the COMPASS Study data portal that the patient was informed over the phone and the brochure was mailed to the patient.

Our protocol and process for consent reflects this integrated model. All stroke patients will be told that the hospital is enrolled in a state-wide initiative to evaluate and improve post-acute stroke care:

- <u>Consent for the Clinical Data (COMPASS Intervention Patients only at 7-14 Day APP Visit)</u>: A signed consent and HIPAA Authorization (collected on the iPad eCare Application) to use data collected from patient during clinical care for research purposes (i.e., to better understand recovery and factors that might influence response to the COMPASS intervention).
- <u>Consent for the 90 day phone survey for patients</u>: A verbal consent over the phone, performed by the UNC Carolina Survey Research Laboratory. COMPASS will rely on the Full HIPAA Waiver for this phone survey, however we have included in the 80 day reminder letter, information on HIPAA for the patient to make an informed decision on if they would like to participating in the phone survey.
- Consent for the 95 day paper survey mailed to caregivers: A returned, completed survey constitutes consent.
- <u>Consent for the Claims Data Analysis</u>: A waiver of consent is requested as this activity (1) is low risk, (2) does not affect the right and welfare of patients, and (3) cannot be practically carried out without this waiver.

Minimal/No Risk Intervention: Participation in the COMPASS study does not expose patients to any additional risks and therefor it is a minimal-risk or no-risk intervention. The COMPASS model and intervention activities are not experimental. COMPASS is evidence-based, and considered best practice for management of post-acute care. CMS supports the delivery of these types of post-acute services and has implemented billing codes (TCM and CCM) to actualize implementation of these services. Hospitals which participate in the COMPASS Study will be asked to implement at the hospital-level these evidence-based services into the systematic delivery of post-acute care to all stroke patients. The COMPASS Study will determine effectiveness of this model on self-reported functional outcomes.

In order to minimize potential differences in loss to follow-up between the control and intervention groups we will send reminder letters (as described in the intervention section) to both control and intervention groups. These letters will include resources from the American Stroke Association (ASA). We will include a refrigerator magnet to remind them that we will call stroke survivors and survey caregivers at 90 days to assess outcomes.

Informed Consent

As a pragmatic trial, our eligibility and outcomes assessment will include all patients discharged home from participating hospitals who are adopting the non-experimental, evidence-based intervention as the new model of care. Our approach has been to minimize patient risk and maximize participation by providing an informational brochure to patients and their families during the hospital stay, and allow them to opt out of the follow-up phone call at 90 days (the primary aim).

Patients will not be asked for consent to participate at the hospital. It is unlikely that our study can seamlessly implement the new models of transitional care in routine clinical care delivery if the traditional informed-consent process for research participation is required. COMPASS will incorporate an Integrated Consent Model for Pragmatic Trials.[31] In addition, during focus groups, patient stakeholders informed us that this would not be the optimal time for informed consent. Patients are often overwhelmed during the hospital stay as they are introduced to a large amount of new information in addition to processing the recent health event (stroke). This was described during the focus group as an emotional and difficult time. Patient stakeholders reported that they are asked to sign a lot of paperwork during the stay and at discharge. According to our stakeholders, the process can be confusing. The COMPASS Study team did not want to add additional burden on the patients and study staff to gain informed consent at the hospital for this low/no risk study.

Figure 3 depicts the flow of Control (left) and COMPASS Intervention (right) activities and how research activities are covered at each step (center).

A HIPAA Waiver which is included as a part of this application is used to confirm eligibility, enrollment, and collect NCSCC Registry Stroke Card data, contact the participants with letters and surveys.

Consent for the Clinical Data (<u>COMPASS Intervention Arm only</u>): At the 7-14 day APP visit, COMPASS participants will be asked for written informed consent and HIPAA Authorization for the study to keep clinical data (2 day phone call, 7-14 day visit, 30 day phone call and 60 day phone call) for future analyses (e.g. follow-up with recommendations for care, demographic and clinical factors that predict

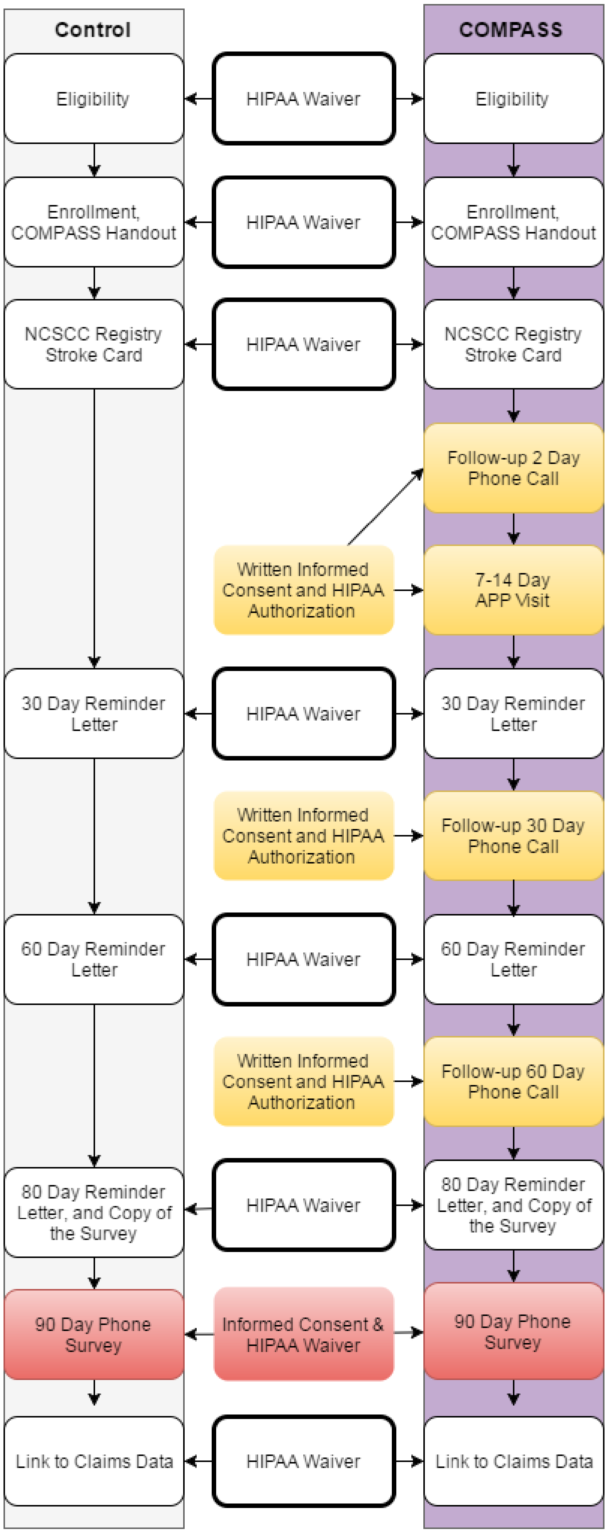

Figure 3: HIPAA Waiver and Consent of COMPASS

follow up and outcomes in the intervention arm). The post-acute coordinator will ask for informed consent and will use the eCare Application on the iPad to capture signature. The abbreviated consent and HIPAA Authorization contains the following elements of informed consent: the purpose of the study; the types of clinical data that will be captured and analyzed; explaining how information the patient provides will be used; data will be kept confidential and secure and will abide to HIPAA regulations; a reminder that providing consent is voluntary; identification of funding agency, study PI, and institution; contact information should the patient want to withdraw (Appendix 21). Patient will use his/her finger to sign and date on the iPad for capture of written signature. (A paper version of the consent form is also available.) The PAC who will be conducting the consent process will also sign and date. A hard copy of consent script will be printed out for the patient to take with them. If the patient declines consent, this will also be noted. Consent will be collected at the end of the visit.

Consent for the 90 Day Phone Survey for Patients (<u>All Patients</u>): All stroke patients (or representing proxies) will consent to (or decline) participation in the survey of outcomes. COMPASS will rely on the Full HIPAA Waiver for this phone survey, however we have included in the 80 day reminder letter, information on HIPAA for the patient to make an informed decision on if they would like to participating in the phone survey. Verbal consent at the introduction of the telephone survey should adequately protect the individuals' rights of patient participants. Study-eligible patients are discharged home, and thus proxy support is unlikely to be needed. If the patient prefers a proxy to complete the survey, the patient can ask the proxy to support them in responding. We will record whether the data are provided by the proxy or the patient.

Consent for the 95 Day Paper Survey Mailed to Caregivers (<u>All Caregivers</u>): With permission of the patient participants, caregivers will be asked to respond to a paper survey questionnaire. Response to the questionnaire will be considered consent and HIPAA Authorization to participate in the study.

Consent for the Claims Data Analysis (<u>All Patients</u>): We will acquire claims data sets using a HIPAA waiver. We will link patient data collected at study enrollment (this data collected is under a HIPAA Waiver) to the claims data. A waiver of consent is requested and included in this application as this activity (1) is low risk, (2) does not affect the right and welfare of patients, and (3) cannot be practically carried out without this waiver.

Consent for Engagement Activities: Consent for research-related engagement activities (i.e. focus groups) will be covered under separate IRBs (PCORI Stakeholder Interviews: IRB00028495; Appendix 31).

Confidentiality and Privacy

Overview

The Principal Investigators and Co-Investigators will ensure the privacy and confidentiality of all study data. All COMPASS Study Investigators and team members are required to complete a yearly HIPAA training and will have training from CITI on Good Clinical Practice Series.

High-levels of security have been put in place to ensure confidential and secure collection, storage and transfer of data. Patient-level data will be stored on secure servers in four different locations, three of which are at UNC-CH and one at Wake Forest Baptist Health:

1. COMPASS Analytic Database housed by (UNC-CH) EMS Performance and Improvement Center (EMSPIC)
2. COMPASS eCare Plan Informatics Database housed by Wake Forest Baptist Medical Center (WFBMC)
3. Carolina Survey Research Lab (UNC-CH) will temporarily store data that is needed to conduct the phone surveys and send reminder letters.
4. Sheps Center (UNC-CH) will support COMPASS and house claims data.

The sections below describe the information technology protections put in place to ensure security of all patient-level data at all times in each database.

COMPASS Analytic Database at EMSPIC

All COMPASS participants will be assigned a unique participant ID that will be used to link participant records and identify participants within the database. Only key study investigators, team members and clinicians will have access to the identity of participants.

A comprehensive Data Use Agreement governs the use of the data collected and stored by the UNC EMS Performance and Improvement Center (EMSPIC) for research purposes.

Data security is achieved through storage in a secure data center (Peak10), data inspection and monitoring (StillSecure), and complex application security. Access to COMPASS data will require three levels of security: a badge and security code to enter EMSPIC, a badge and fingerprint scan to access the data center, and a security code for each data rack. All outside access to servers and databases must be accomplished through a Virtual Private Network (VPN). All EMSPIC applications use a strong and sophisticated security module, which restricts access based on entity assignments, and security rights monitored by EMSPIC staff. All applications are only accessible via Hypertext Transfer Protocol Secure (HTTPS). HTTPS is a layering of the standard internet protocol (HTTP) onto an SSL (Secure Sockets Layer) protocol. This results in bidirectional encryption of communications between the client and server and serves as reasonable security against eavesdropping on or tampering with the contents of that communication. The HTTPS protocol will be used for all application through SSL hardware encryption and signed by an accepted root certificate authority.

EMSPIC does not allow the use of portable storage devices and unencrypted data will never be stored on flash drives, external hard disks, or laptops. All applications developed at the EMSPIC

prevent SQL Injection attacks from occurring by isolating all form data by escaping incoming string data.

Electronic Care (eCare) Plan Informatics Database

The COMPASS eCare Plan Application is a secure web-based application created by a HIPAA-trained programming team at Wake Forest Baptist Medical Center to capture intervention-related data. As described in the intervention section, the eCare Plan Application supports health care providers in efficiently and systematically evaluating patients and identifying next steps for referrals. Data collected into this application will be used to support providers in creation of an individualized care plan (eCare Plan) for patients and generate referral note(s) to other providers (if needed).

The eCare Plan Application is a secure application utilizing TLS level security (a more secure version of SSL). Communication and data transfer between the user's device (iPad, Desktop, Laptop) and eCare Plan Application are encrypted at all times. To access the eCare Plan Application, health care providers must user-authenticate into the COMPASS portal. The eCare Plan Application will employ role-based security which will limit users access to only information they were authorized to access. . Users will be asked to change their password regularly. Data will not be stored locally on any devices to minimize the risk associated with any lost or stolen devices.

The eCare Plan Informatics Database is part of a SQL Server relational data warehouse which is housed in the Wake Forest Health Sciences A1a data center on 3rd Street in Winston-Salem, NC. The webserver hosting the eCare Plan Application is also hosted in the A1a data center. The webserver is a virtual server so in the event of disaster or unexpected significant and lengthy interruption, we can migrate the server into a second data center on Miller St in Winston-Salem, NC . The servers are contained within a secure data center with environmental controls which detect abnormal conditions such as power outages, high heat or humidity, and loud sound. The A1a data center has several secure access points that are accessible only by a badge reader. Only authorized staff will have access to these areas. The building is surrounded by a 10-foot fence with a gate access through badge control. The outside building door is accessed through badge control. The data center room is housed in a locked computer room that is accessed through badge control. Each of these access controls is in place 24 hours a day and seven days a week. All servers have uninterruptible power supplies (UPS). The building has a backup generator that will automatically initiate in the event of a power failure. The computer room is equipped with fire suppression equipment. This equipment is tested on a scheduled timetable by the institution. The entire Data Center is fire- protected by a clean agent system which is backed up by a dry-pipe pre-action sprinkler system. The Data Center room is located on the second floor of the building in an area with no windows and has a raised floor to protect against flooding.

Carolina Survey Research Lab Database and Security

Staff at the CSRL must complete training on Human Subjects Protection, Conflict of Interest, and sign a Confidentiality Agreement. The team is provided a wide variety of computing resources for data collection, statistical computing, and office automation. Staff members and

research assistants are provided with a Pentium class microcomputer running Windows 7 and Microsoft Office Professional, as well as SAS 9.3 and SUDAAN 10.0 for statistical analysis, virus detection software, and a wide variety of other standard microcomputer software.

CSRL computers communicate securely through the UNC- ITS systems for Internet communications and for access to secure files which have automatic back-up. All sensitive information is hosted on a server that meets the University Information Security policy (http://its.unc.edu/files/2014/08/Information-Security-Policy.pdf). This policy includes, but is not limited to, the following configurations: host based and network based firewalls, least functionality, least privileged, weekly vulnerability scans, secure backup (located in a separate location on campus), secured physical access, password enforcement policy, warning banner, incident management plan, monitored malware protection, and patch management.

CSRL manages information in a variety of forms including paper, diskettes, and electronic databases. The CSRL maintains a secure file room in an interior room within a suite for the storage of original paper forms and sensitive data on diskettes. This room is locked at all times and only select staff have access to it. For electronic databases, the CSRL employs two servers: (1) data collection machines, which may collect personal identifiers, are protected on a server behind a physical firewall that is cut-off from the Internet; and (2) data analysis machines have access to a server that stores de-identified data; that is, data collected through the calling room machines that have been stripped of any potential identifiers.

To facilitate surveys, patient and caregiver names, phone numbers and addresses, date of hospital discharge, preferred day and time of contact, as well as PAC information will be exported as a CSV file from the Analytic Database. This file will be stored on a secure server (\\cecil.schsr.unc.edu) and accessed by staff at CSRL for pre-loading into their phone system once per week. All access to this secure server will only be granted through UNC secure VPN.

Trained and approved staff at CSRL will have access to COMPASS Analytic Database to record responses and avoid storing patient level-data in a database outside of the primary COMPASS database.

Sheps Center Data Security

The Sheps Center will be housing administrative claims data and the server to which EMSPIC and CSRL will post their shared files. Claims data files at the Sheps Center are placed on a secure dataset server configured specifically to handle large-scale health utilization data. Each data file has access restricted to those users authorized by the relevant DUA. The primary dataset directories on the dataset server provide one inventory of our current claims data files. Disk-to-disk backups of claims data files on our dedicated dataset server are made nightly to two separate backup servers at two different Data Center locations on the UNC campus.

Claims data files are housed on a dedicated secure dataset server configured specifically for sensitive health utilization data. The server is physically located in a Tier II data center at 440 W Franklin St, Chapel Hill as part of the UNC campus. These facilities have 24x7 surveillance, multiple power sources and backup power sources, climate control, etc. Our systems

administrators get access via electronic pass cards. Each card entry gets recorded with time/date stamps in facility access logs. The dataset server is behind a firewall, accessible only to Sheps Center IP addresses and UNC Secure VPN addresses. There is no printer attached to the dataset server. Only aggregated (anonymized) data and SAS output are taken off the server and onto local computers.

The server is a Linux (RedHat enterprise) server with all unneeded services disabled. Access to the claims data on the Sheps Dataset Server will be restricted to users authorized by the PI. The server is routinely patched with system updates and receives twice-weekly vulnerability scans using Qualys. Identified vulnerabilities are addressed according to UNC Security Policy. Access to the server is via SSH. Off campus access is restricted to UNC VPN connections requiring a user to authenticate with VPN before server login. VPN also provides an encrypted tunnel. SAS and Stata are typical data programs used.

The Sheps Center makes two daily disk-to-disk backups of the secure dataset server. One backup goes to a dedicated backup server within the same Tier II data center at 440 W Franklin. The second goes to a second dedicated backup server at a second Tier II data center located across town at 211 Manning Drive, Chapel Hill. The backup data travel via an SSH tunnel over the same VLAN within the UNC campus firewalls. Both backup servers are behind a firewall denying the ability of other computers or servers to initiate a connection to the backup servers. Instead, the backup servers reach out to the secure dataset server and “pull” the backup data over.

The hard drives and CDs on which data have been delivered will be stored in a locked cabinet at the Sheps Center. Only authorized staff will have keys to the cabinet. The office will be locked when not occupied. In addition, the Sheps Center is locked 24 hours a day.

User level access to claims data files is restricted based on authorized roles. Unix groups are leveraged to provide layered controls. Users may access the data in the following ways:

1. Using a computer that is on the UNC Active Directory domain and is managed by UNC ITS security tools that perform required scans for viruses and malware and force updated software and operating system patches. Users with this type of computer (desktop or laptop) can access the server directly from campus or via a remote VPN using SSH.
2. Using a computer not managed by UNC ITS security tools via a specifically designated secured UNC Virtual Computer (virtual computing lab), which connects to the server. This virtual desktop is setup, maintained, and managed by Sheps Center sys admins. At the end of a working session, the virtual computer is destroyed along with any data that may have been used locally in the virtual computer instance.

Logical access is safeguarded at multiple levels:

1. The claims data files will be housed on a dedicated secure dataset server configured specifically for sensitive health utilization data. The server is physically located in a Tier II data center at 440 W Franklin St, Chapel Hill, an extension of the UNC campus. These facilities are governed by the UNC ITS Data Center Operations policies and procedures. They have 24x7 surveillance, required visitor sign-in with escorts, multiple power sources and backup power sources, climate control, etc. Our systems administrators get access via electronic pass cards. Each card entry gets recorded with time/date stamps in

facility access logs. While other sys admins of ITS have access to the same physical space where our server racks are housed, these are trusted people governed by central campus ITS policies and procedures. Also, the Data Center space has logged entry of individuals and is under constant surveillance where any unauthorized physical access would be monitored and recorded. Significant sanctions are known and apply, up to and including job termination and possible criminal prosecution.

2. The designated server is firewalled allowing only SSH port 22 to be open. All unnecessary ports are closed and all unnecessary services are disabled. User level access to files is restricted based on authorized roles. SSH connections are limited to UNC subnets and UNC VPN addresses. Access to the designated secure dataset server is restricted to computers within the UNC network domain. Connections originating outside the UNC network are restricted to UNC VPN authentication first and then system-level user/pw authentication. Users may only connect using SSH and Kerberos authentication leveraging the UNC Single Sign-on policies and procedures.
3. Nightly secure backups are performed to two dedicated backup servers – one backup within the same data center within the firewalled subnet and the second backup in another data center across town via the same VLAN. The original CMS data is not backed up to tape but instead the delivery media is kept for backup, if needed. In case of (a) data center disaster, or (b) backup failure, a second backup computer is housed in a second campus data center. Two system administrator computers are allowed to connect to backup servers to control them. Firewall prevents all other computers from reaching backup servers. Backup computers initiate the network connections to the server. It is not possible for any user to initiate a connection to a backup server from the main server or from any other computer except for those owned by two system administrators. All network connections are encrypted.
4. The designated server is kept up-to-date with recommended operating system patches and patches for applications. The server is scanned twice weekly for vulnerabilities using the UNC licensed QualysGuard SaaS software. System administrators monitor event logs, security logs, and system logs. UNC uses Snort for intrusion detection and Tipping Point for intrusion prevention. These systems/appliances are monitored and handled centrally by the UNC ITS Security Office. Suspicious activity is reported to the Sheps Center's Security Liaison for investigation and handling with assistance from the central ITS Security Office. If these data will be delivered via CD or hard drive, the media will be kept in a locked storage location provided by the project, with key access only to research team members.
5. Project staff at the Sheps Center will access the secure dataset server via SSH using computers physically in the Sheps Center Building. The Sheps Center Building's exterior doors are locked 24x7. Individual offices inside the Sheps Center are also locked. Staff enter using an authorized key. Visitors must be buzzed in using a video intercom system and then must report to the receptionist and sign in. There are no servers physically located in the Sheps Center Building.

Description of the Secure Data Transfers

Secure Data Transfer between the COMPASS DB and eCare Plan Informatics (eCPI) DB
Data transfer between the COMPASS Database and the eCPI Database will be via RESTful web services designed specifically for the limited datasets being exchanged. Secure data transfer protocols will be in place to provide fully encrypted transmissions. The eCare Application may retrieve and update patient data from the COMPASS Database (the database of record for patient data) for use during a patient visit in order to foster the use of that data in creating an eCare Plan. Patient data is requested based on key identifiers and the resulting match reflected in the eCare application. The web service will allow for the transmission of data stored in the eCPI database to the COMPASS Database for use in analysis as well as patient status updates and notifications to appropriate study personnel. The eCare database remains the database of record for those intervention data while making the COMPASS DB and associated application aware of the analytical data needed for the study. The eCare application will contain the logic necessary during the flow of its data collection to validate data with the patient as well as prevent implausible and/or out-of-range responses.

Secure Data Transfer between COMPASS DB and CSRL
As described above, to facilitate surveys, patient and caregiver names, phone numbers and addresses, date of hospital discharge, preferred day and time of contact, as well as PAC information will be exported as a CSV file from the Analytic Database. This file will be stored on a secure server (\\cecil.schsr.unc.edu) and accessed by staff at CSRL for pre-loading into their phone system once per week.

Data Access for Analysis

We have in place secure operations for sharing SAS datasets between investigators to ensure safety and confidentiality of patients. Datasets will be stored on a secure server that is accessed through a virtual machine. Study investigators will be granted access to this server for running data reports and analyses of the study data. The files will be read-only and analyses will be conducted through a virtual machine so that data are never temporarily stored on or transferred to a user's computer. These internal datasets will contain the COMPASS Unique participant IDs to link data in different files together. PHI (including date of birth, medical record number, names, addresses, phone numbers, and email addresses) will be removed from datasets prior to export of these SAS datasets from the COMPASS Database.

REDCap (Research Electronic Data Capture)

COMPASS Study will use REDCap at Wake Forest Baptist Medical Center to store responses from hospital stakeholders and track stakeholder engagement activities.

Vanderbilt University, with collaboration from a consortium of institutional partners, has developed a software toolset and workflow methodology for electronic collection and management of research and clinical trial data. REDCap (Research Electronic Data Capture) is hosted at Wake Forest Baptist Medical Center through the Biomedical Informatics program of the Translational Science Institute. REDCap servers are located within the Wake Forest Baptist Health firewall and all web-based information transmission is SSL (Secure Socket Layer) encrypted; the databases are backed up nightly through the institution's enterprise backup

system. Users are granted access to the system using their unique medical center username and password with specific access rights setup for each study. REDCap was developed specifically around HIPAA-Security guidelines and is used by 1,000+ academic/non-profit consortium partners on six continents with over 195,000 research end-users (www.project-redcap.org).

Data and Safety Monitoring

The COMPASS Data and Safety Monitoring Board (DSMB) will serve to support as an independent review board of the study and study activities to protect patients. Adverse events among this patient population are likely; however because COMPASS is a minimal/no risk study, COMPASS will not be tracking adverse events. Therefore the COMPASS DSMB will provide annual review of study activities providing external review and assurance on the performance of the study. This annual review will include case ascertainment and enrollment; expected versus observed outcomes; review of percentage of participants in the intervention receiving the 2-day call, the 7-14 day visit, the eCare Plan; and review of protocol deviations.

To avoid any appearance of conflict of interest, it is critical that DSMB members not be involved in the study, have no vested interest in its outcome, have no ties to the study investigators (e.g., not from the same institution and no history of extensive collaboration), and have no financial ties to any commercial concerns likely to be affected by the study's outcome. If at any time a DSMB member perceives that he/she or another member of the Board has a potential conflict of interest, he/she is obligated to bring the issue to the attention of the full DSMB for open discussion and resolution.

Responsibilities

1. COMPASS DSMB members will be responsible for assuring study participants are not exposed to unnecessary, unreasonable or unexpected risk, and is charged with ensuring that the study is conducted according to the highest scientific and ethical standards.
2. Specifically, oversight will include the following areas:
    - Review of the COMPASS Manual of Operations and Procedures (MOP), the analysis plan, and implementation of the study procedures at the first DSMB meeting.
    - Review of study protocol including our informed consent processes.
    - The DSMB may recommend modifications or request clarifications of the protocol.
    - Review of the study outcomes and their clear definition, study procedures, informed consent documents, data security, and investigator responsibilities.
    - In subsequent meetings, the DSMB will focus on case ascertainment and enrollment; expected versus observed outcomes; review of percentage of participants in the intervention receiving the 2-day call, the 7-14 day visit, the eCare Plan; and review of protocol deviations.
    - Any other areas the DSMB considers oversight to be necessary.

Frequency of Meetings and Communication between DSMB and COMPASS

COMPASS DSMB members will meet annually. The first meeting will take place in person, in early 2016. Subsequent meetings will take place remotely over webinar. Meetings will be closed to the public. Only DSMB members and members of the COMPASS Executive Leadership Committee will attend. ELC members will prepare in advance a DSMB report for review (Appendix 32). Each meeting will start with discussion between COMPASS Executive Leadership Team and DSMB and then the DSMB will meet privately without study personnel.

At the end of each annual meeting, the DSMB will provide a verbal report to the Executive Leadership Team noting any areas of concern in study performance and/or operations. Care will be exercised to ensure no information will be conveyed that could compromise the study or its outcomes. Within two weeks, the DSMB Chair will provide a written report to PCORI and the Executive Leadership Team, which includes the DSMB recommendation for continuing, discontinuing, amending, or suspending the study. This written report will cover data reviewed, recommendations, and date of the next scheduled

review. This report will be forwarded by the PIs to the Central IRB at Wake Forest Medical Center and the Data Coordination IRB at the University of North Carolina and PCORI.

Membership

The process for identifying the DSMB included feedback from PCORI, recommendations from the Steering Committee and vetting by the Executive Leadership Committee (ELC). The ELC reviewed each recommendation and consulted with PCORI for additional guidance and input on the final selection of DSMB members. Once DSBM members were approved by the ELC, the Project Manager sent out a formal letter to the proposed DSMB members inviting them to serve. COMPASS DSMB members include:

DSMB Chair:

1. Jason Conner, PhD – Director and Senior Statistical Scientist for Berry Consultants. Dr. Connor has expertise in Bayesian statistics and designing adaptive clinical trials. He serves on the Clinical Trials Advisory Panel (CTAP) for PCORI. Dr. Connor has accepted to serve as a DSMB member.

DSMB Members:

2. Judy Lichtman, PhD, MPH – Chair, Chronic Disease Epidemiology, Yale University. Dr. Lichtman focuses on stroke research and is experienced in outcomes research, quality improvement and CMS data linkage. Dr. Lichtman has accepted to serve as a DSMB member.
3. Brett Kissela, MD – Chair of Department of Neurology and Rehabilitation Medicine at the University of Cincinnati (UC) College of Medicine and UC Health. Dr. Kissela is a stroke neurologist and stroke researcher. Dr. Kissela has accepted to serve as a DSMB member.
4. Theresa Damush, PhD – Associate Research Professor of Medicine, Indiana University School of Medicine. Dr. Damush is a research health psychologist focusing on implementing evidence-based practices for stroke survivors and caregivers. She specializes in the design and evaluation of patient centered programs. Dr. Damush has accepted to serve as a DSMB member.

Reporting of Unanticipated Problems, Adverse Events or Deviations

Any unanticipated problems, deviations or protocol changes will be promptly reported by the principal investigator or designated member of the research team to the IRB and sponsor or appropriate government agency if appropriate.

References

1. American Heart Assosiation. Heart Disease and Stroke Statistics—2015 Update. *Circulation*. 2015;131:e29–e322.
2. Bertoni AG, Ensley D, Goff DC. 30,000 fewer heart attacks and strokes in North Carolina: a challenge to prioritize prevention. *N. C. Med. J.* 2012;73:449–456.
3. Kennedy BS. Does race predict stroke readmission? An analysis using the truncated negative binomial model. *J. Natl. Med. Assoc.* 2005;97:699–713.
4. Centers for Medicare and Medicaid Services. Chronic Conditions among Medicare Beneficiaries. Baltimore, MD: 2012.
5. Olson DM, Cox M, Pan W, Sacco RL, Fonarow GC, Zorowitz R, LaBresh KA, Schwamm LH, Williams L, Goldstein LB, Bushnell CD, Peterson ED. Death and Rehospitalization after Transient Ischemic Attack or Acute Ischemic Stroke: One-year Outcomes from the Adherence Evaluation of Acute Ischemic Stroke–Longitudinal Registry. *J. Stroke Cerebrovasc. Dis.* 2013;22:e181–e188.
6. Fonarow G, Smith E, Reeves M, Pan W, Olson D, Hernandez A, Peterson E, Schwamm L. Hospital-level variation in mortality and rehospitalization for Medicare beneficiaries with acute ischemic stroke. *Stroke*. 2011;42:159–166.
7. Rosamond W, Johnson A, Bennett P, O'Brien E, Mettam L, Jones S, Coleman S. Monitoring and improving acute stroke care: The North Carolina Stroke Care Collaborative. *N. C. Med. J.* 2012;73:494–498.
8. Bernhardt J, Dewey H, Thrift A, Donnan G. Inactive and Alone Physical Activity Within the First 14 Days of Acute Stroke Unit Care. *Stroke*. 2004;35:1005–1009.
9. Bamford J, Dennis M, Sandercock P, Burn J, Warlow C. The frequency, causes and timing of death within 30 days of a first stroke: the Oxfordshire Community Stroke Project. *J. Neurol. Neurosurg. Psychiatry*. 1990;53:824–829.
10. Johnston KC, Li JY, Lyden PD, Hanson SK, Feasby TE, Adams RJ, Faught RE, Haley EC. Medical and Neurological Complications of Ischemic Stroke Experience From the RANTTAS Trial. *Stroke*. 1998;29:447–453.
11. Whitson HE, Pieper CF, Sanders L, Horner RD, Duncan PW, Lyles KW. Adding injury to insult: fracture risk after stroke in veterans. *J. Am. Geriatr. Soc.* 2006;54:1082–1088.
12. Davenport RJ, Dennis MS, Wellwood I, Warlow CP. Complications after acute stroke. *Stroke J. Cereb. Circ.* 1996;27:415–420.
13. White JH, Attia J, Sturm J, Carter G, Magin P. Predictors of depression and anxiety in community dwelling stroke survivors: a cohort study. *Disabil. Rehabil.* 2014;:1–8.
14. Husseini NE, Goldstein LB, Peterson ED, Zhao X, Pan W, Olson DM, Zimmer LO, Williams JW, Bushnell C, Laskowitz DT. Depression and Antidepressant Use After Stroke and Transient Ischemic Attack. *Stroke*. 2012;43:1609–1616.
15. Kelly J, Rudd A, Lewis RR, Coshall C, Moody A, Hunt BJ. Venous Thromboembolism After Acute Ischemic Stroke A Prospective Study Using Magnetic Resonance Direct Thrombus Imaging. *Stroke*. 2004;35:2320–2325.
16. Lai S-M, Studenski S, Duncan PW, Perera S. Persisting Consequences of Stroke Measured by the Stroke Impact Scale. *Stroke*. 2002;33:1840–1844.
17. Bushnell CD, Zimmer LO, Pan W, et al. Persistence with stroke prevention medications 3 months after hospitalization. *Arch. Neurol.* 2010;67:1456–1463.
18. Kopunek SP, Michael KM, Shaughnessy M, Resnick B, Nahm E-S, Whitall J, Goldberg A, Macko RF. Cardiovascular Risk in Survivors of Stroke. *Am. J. Prev. Med.* 2007;32:408–412.
19. Rigby H, Gubitz G, Eskes G, Reidy Y, Christian C, Grover V, Phillips S. Caring for stroke survivors: baseline and 1-year determinants of caregiver burden. *Int. J. Stroke Off. J. Int. Stroke Soc.* 2009;4:152–158.

20. Rigby H, Gubitz G, Phillips S. A systematic review of caregiver burden following stroke. *Int. J. Stroke*. 2009;4:285–292.
21. Prvu Bettger J, Alexander KP, Dolor RJ, Olson DM, Kendrick AS, Wing L, Coeytaux RR, Graffagnino C, Duncan PW. Transitional Care After Hospitalization for Acute Stroke or Myocardial InfarctionA Systematic Review. *Ann. Intern. Med.* 2012;157:407–416.
22. Fearon P, Langhorne P, Early Supported Discharge Trialists. Services for reducing duration of hospital care for acute stroke patients. *Cochrane Database Syst. Rev.* 2012;9:CD000443.
23. Langhorne P, Taylor G, Murray G, Dennis M, Anderson C, Bautz-Holter E, Dey P, Indredavik B, Mayo N, Power M, Rodgers H, Morten Ronning O, Rudd A, Suwanwela N, Widen-Holmqvist L, Wolfe C. Early supported discharge services for stroke patients: a meta-analysis of individual patients' data. *Lancet*. 2005;365:501–506.
24. Kernan WN, Ovbiagele B, Black HR, Bravata DM, Chimowitz MI, Ezekowitz MD, Fang MC, Fisher M, Furie KL, Heck DV, Johnston SC (Clay), Kasner SE, Kittner SJ, Mitchell PH, Rich MW, Richardson D, Schwamm LH, Wilson JA. Guidelines for the Prevention of Stroke in Patients With Stroke and Transient Ischemic Attack A Guideline for Healthcare Professionals From the American Heart Association/American Stroke Association. *Stroke*. 2014;:STR.0000000000000024.
25. Teasell R. Evidence-Based Review of Stroke Rehabilitation [Internet]. [cited 2014 Jun 14];Available from: http://www.ebrsr.com/
26. Legg LA, Quinn TJ, Mahmood F, Weir CJ, Tierney J, Stott DJ, Smith LN, Langhorne P. Non-pharmacological interventions for caregivers of stroke survivors. *Cochrane Database Syst. Rev.* 2011;:CD008179.
27. Forster A, Brown L, Smith J, House A, Knapp P, Wright JJ, Young J. Information provision for stroke patients and their caregivers. *Cochrane Database Syst. Rev.* 2012;11:CD001919.
28. Salter K, Allen L, Richardson M, Teasell R. Community Reintegration [Internet]. EBRSR.COM; 2013. Available from: http://www.ebrsr.com/sites/default/files/Chapter19_Community-reintegration_FINAL_16ed.pdf
29. Tu J, Willison D, Silver F, Fang J, Richards J, Laupacis A, Kapral M. Impracticability of informed consent in the Registry of the Canadian Stroke Network. *New Engl J Med*. 2004;350:1414–1421.
30. Centers for Medicare & Medicaid Services. Hospital Compare Datasets. [https://data.medicare.gov/data/hospital-compare]
31. Kim, SYH and Miller FG. Informed Consent for Pragmatic Trials — The Integrated Consent Model. N Engl J Med 2014; 370:769-772

Appendices
